# Synthesis of the Double Infinite-Layer Ni(I) Phase La_3_Ni_2_O_5_F via Sequential Topochemical Reactions

**DOI:** 10.1021/jacs.5c16740

**Published:** 2026-02-04

**Authors:** Romain Wernert, Robert D. Smyth, Michael A. Hayward

**Affiliations:** Department of Chemistry, Inorganic Chemistry Laboratory, University of Oxford, South Parks Road, Oxford OX1 3QR, United Kingdom

## Abstract

Fluorination of the *n* = 2 Ruddlesden–Popper oxide, La_3_Ni_2_O_7_, with polyvinylidene fluoride yields La_3_Ni_2_O_5_F_4_, a phase in which fluoride ions have been inserted into interstitial sites in the Ruddlesden–Popper framework and also exchanged with the oxide ions residing on apical anion sites. Reaction with LiH at 190 °C reduces La_3_Ni_2_O_5_F_4_ by extracting interstitial fluoride ions. The resulting phase, La_3_Ni_2_O_5_F_3_, adopts a structure described in space group *Pbcm* in which the fluoride ions in the half-filled interstitial layer are arranged in chains parallel to the *y*-axis, and the NiO_5_F octahedra adopt an *a* ^–^ *a* ^–^ *c* ^+^ */–(a* ^–^ *a* ^–^ *)­c* ^+^ tilting pattern. Further reduction with LiH at 250 °C converts La_3_Ni_2_O_5_F_3_ into La_3_Ni_2_O_5_F, a Ni^1+^ phase which adopts a T′-structure consisting of double infinite-sheets of apex linked NiO_4_ squares, stacked with LaOF fluorite-type layers. Magnetization and neutron diffraction data indicate La_3_Ni_2_O_5_F_3_ adopts an antiferromagnetically ordered state below *T*_N_ = 225 K, while magnetization data from La_3_Ni_2_O_5_F exhibit a broad maximum centered at 75 K, suggestive of antiferromagnetic order.

## Graphical abstract

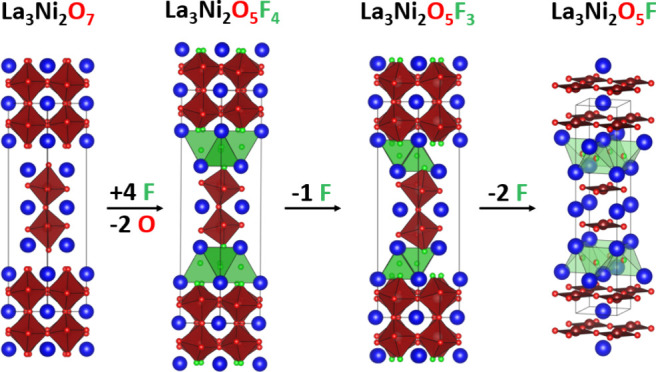

## Introduction

Extended solids containing transition-metal cations exhibit a wide range of complex electronic and magnetic behaviors which arise from the presence of electrons in partially occupied d-states and bands. The interactions between these d-electrons are strongly dependent on both the crystal structure and composition of the host solid, and as a result the complex correlated electronic behavior of transition-metal containing solids can be modified and tuned by chemical means.^1,2^

The high-temperature superconductivity observed in complex copper oxides is a good example of such behavior. In these systems superconductivity is typically induced by taking a Cu­(II) oxide, such as La_2_CuO_4_, and removing electrons (hole doping)^3^ from the copper d-states via chemical substitution, to disrupt the antiferromagnetic ground state of the undoped material. In the majority of cuprates the superconducting transition temperature, *T*_c_, is maximized at doping levels of ∼ 0.15 electrons per Cu.^4^ In the case of La_2_CuO_4_ this doping level can be achieved either by A-site substitution (i.e., La_2‑x_Ba_*x*_CuO_4_)^3^ or insertion of additional anions into the interstitial sites within the rock salt layers of the *n* = 1 Ruddlesden–Popper framework (i.e., La_2_CuO_4+x_),^5,6^ as substitution on the copper B-site (i.e., La_2_Cu_1–*x*_M_*x*_O_4_) rapidly suppresses superconductivity.^7–9^

The isoelectronic relationship between d^9^ Cu­(II) oxides and systems containing Ni­(I)­O_4_ units led to predictions of analogous superconducting behavior in infinite-layer nickel oxides.^10^ However, it was not until the preparation of thin-film samples of the hole-doped, infinite-layer nickelate Nd_0.8_Sr_0.2_NiO_2_ that superconductivity was observed in such a system.^11^ Superconductivity has also been observed in thin-film samples of the quintuple-layer phase Nd_6_Ni_5_O_12_ (prepared via reduction of Nd_6_Ni_5_O_18_) which has the same average Ni oxidation state (Ni+1.2) as Nd_0.8_Sr_0.2_NiO_2_.^12^ However, as yet superconductivity has not been observed in other members of the *Ln*_*n*+1_Ni_*n*_O_2n+2_ series (*Ln* = rare earth), of which Nd_0.8_Sr_0.2_NiO_2_ and Nd_6_Ni_5_O_12_ represent the *n* = ∞ and *n* = 5 members, respectively. Presumably this is because *n* = 2 and *n* = 3 phases such as La_3_Ni_2_O_6_ or La_4_Ni_3_O_8_ have Ni oxidation states of Ni+1.5 and Ni+1.33 respectively,^13,14^ which are too high to support superconductivity, and these phases cannot be easily electron doped by cation exchange.

Recently we have demonstrated that the regioselectivity of topochemical reduction reactions can be modified by using a fluorinate-then-reduce synthetic strategy.^15^ Here we report the application of this reaction sequence to La_3_Ni_2_O_7_, to prepare La_3_Ni_2_O_5_F, an electron-doped version La_3_Ni_2_O_6_.

## Experimental Section

### Solid-State Synthesis

A 3 g sample of La_3_Ni_2_O_7_ was prepared by a citrate gel method, followed by a high temperature annealing. Suitable stoichiometric ratios of metallic Ni (99.99%) and La_2_O_3_ (99.999%, dried at 900 °C) were dissolved in a minimum amount of 1:1 deionized water: HNO_3_. Citric acid and ethylene glycol were added to complex the metal ions and the excess water was evaporated until a gel formed, which was heated further until a black powder remained. The powder was then heated in air at 500 °C for 12 h, to decompose the remaining organic residues, ground again and then pressed into pellets prior to being heated to 1150 °C under oxygen flow for two periods of 48 h, until no further change was observed in powder X-ray diffraction data. Diffraction data collected from the final product confirmed a single-phase powder and could be readily fitted by a model of the reported structure of La_3_Ni_2_O_7_ described in space group *Amam* and cell parameters *a* = 5.3914(2) Å, *b* = 5.4467(2) Å, *c* = 20.5186(6) Å.^16^ The standard samples of La_2_NiO_4_, LaNiO_3_ and LaNiO_2_ were synthesized according to previous reports as detailed in the Supporting Information.^17,18^

### Fluorination of La_3_Ni_2_O_7_

Fluorination of La_3_Ni_2_O_7_ was performed using polyvinylidene fluoride powder (PVDF, Fluorochem). Two grams of La_3_Ni_2_O_7_ and 1 g of PVDF were put in two separate alumina boat crucibles which were placed next to each other within a tube furnace, arranged so that oxygen would flow first over the PVDF and then the La_3_Ni_2_O_7_ sample. The apparatus was then heated to 310 °C for 12 h. This treatment was repeated twice, after which no further change was observed in the powder X-ray diffraction data collected from the product. Finally, the sample was annealed in a sealed, evacuated borosilicate tube at 340 °C for 12 h to improve crystallinity.

### Topochemical Reduction

Samples of fluorinated La_3_Ni_2_O_7_ were reduced by reaction with LiH. Test reactions to assess reactivity were performed on small samples (∼250 mg) which were ground together with 6 mol equivalents of LiH in an argon-filled glovebox. The mixtures were sealed within evacuated borosilicate glass tubes and heated for periods of 2 days at temperatures between 150 °C (no reaction) and 275 °C (decomposition). Larger samples (∼1.25 g) used for subsequent analysis were prepared as described above using larger ampules to minimize H_2_ pressure build up. Following reaction, LiH-reduced samples were washed with methanol to remove unreacted LiH and the Li_2_O impurities before being dried under vacuum and stored in an Ar-filled glovebox.

### Characterization

Reaction progress was monitored and initial structural characterizations were performed using X-ray powder diffraction data collected using a Bruker D8 Advance diffractometer equipped with a LYNXEYE silicon strip detector and operating with Cu K_α_ radiation. Air sensitive samples were measured in airtight sample holders under argon. High resolution synchrotron powder X-ray diffraction (SXRD) data were collected using the I11 instrument at Diamond Light Source, using Si-calibrated X-rays of approximate wavelength 0.824 Å and a Mythen 3 position sensitive detector. Samples were diluted in ground silica glass to minimize absorption and then sealed 0.5 mm diameter borosilicate glass capillaries. Neutron powder diffraction (NPD) data were collected using the POLARIS diffractometer at the ISIS neutron and muon source.^19^ Samples were contained in 6 mm diameter vanadium cans sealed under Ar with an indium gasket. Rietveld refinements against diffraction data were performed using the TOPAS Academic v7 software package.^20^ X-ray absorption spectroscopy data were collected using beamline B18 at the Diamond Light Source.^21^ The measurements were carried out using the Pt-coated branch of the collimating mirrors with Si(111) monochromator. Appropriate amounts of sample to achieve an absorption length of 1.6 were mixed with 70 mg of cellulose and pressed into 13 mm diameter pellets. The discs were taped on the sample holder which was subsequently sealed in a plastic bag under argon. DC magnetization data were collected using a Quantum Design MPMS-3 SQUID magnetometer from samples contained in gelatin capsules. Iodometric titration was used to determine the average oxidation state of the transition metals within samples. Precisely weighed sample portions (∼30 mg) were dissolved in 2:1 HCl:deionized water in the presence of excess KI (∼300 mg) and the liberated I_2_ was titrated against a standardized Na_2_S_2_O_3_ solution with a starch indicator being added as the end point approached. A constant argon flow was maintained during the titration to avoid oxidation by air.

## Results

### Structural and Chemical Characterization of La_3_Ni_2_O_5_F_4_

Synchrotron X-ray powder diffraction data collected from the fluorination product of La_3_Ni_2_O_7_ could be readily indexed using an orthorhombic unit cell (*a* = 5.413 Å, *b* = 5.492 Å, *c* = 22.418 Å). These data were observed to be very similar to a previously reported phase, La_3_Ni_2_O_5.5_F_3.5_ (*a* = 5.431 Å, *b* = 5.507 Å, *c* = 22.483 Å), which was prepared from La_3_Ni_2_O_7_ using CuF_2_ as a fluorinating agent.^22^ A structural refinement against the SXRD data using the latter structure as a starting model gave a good statistical fit (Figures S3 and S4 and Tables S1 and S2). However, in contrast to the CuF_2_-prepared sample reported previously,^22^ an anion composition of La_3_Ni_2_O_5_F_4_ was determined for the PVDF-prepared phase by charge balancing the chemical formula La_3_Ni_2_O_7–2*x*_F_2+2*x*_ (0 ≤ *x* ≤ 1) to the oxidation state of nickel (determined to be Ni^2.5+^ from Ni K-edge X-ray absorption spectroscopy (Figure S5) and Ni^2.54(3)+^ by iodometric titration). This composition was confirmed by TGA and annealing measurements as described in the Supporting Information. Finally, the oxide/fluoride distribution was established by calculating the bond valence sums (BVS) of the anion sites using the bond valence parameters for O^2–^ or F^–^ (Table S3). It can be observed from these calculations that the BVS of the apical and interstitial sites are well below the expected value of 2 if considered as O^2–^ ions, in contrast to the bridging and equatorial sites, indicating that the fluorination of La_3_Ni_2_O_7_ with PVDF fills the interstitial site with fluoride ions and entirely substitutes the apical site with fluoride ions as shown in Figure 1.

**Figure 1:**
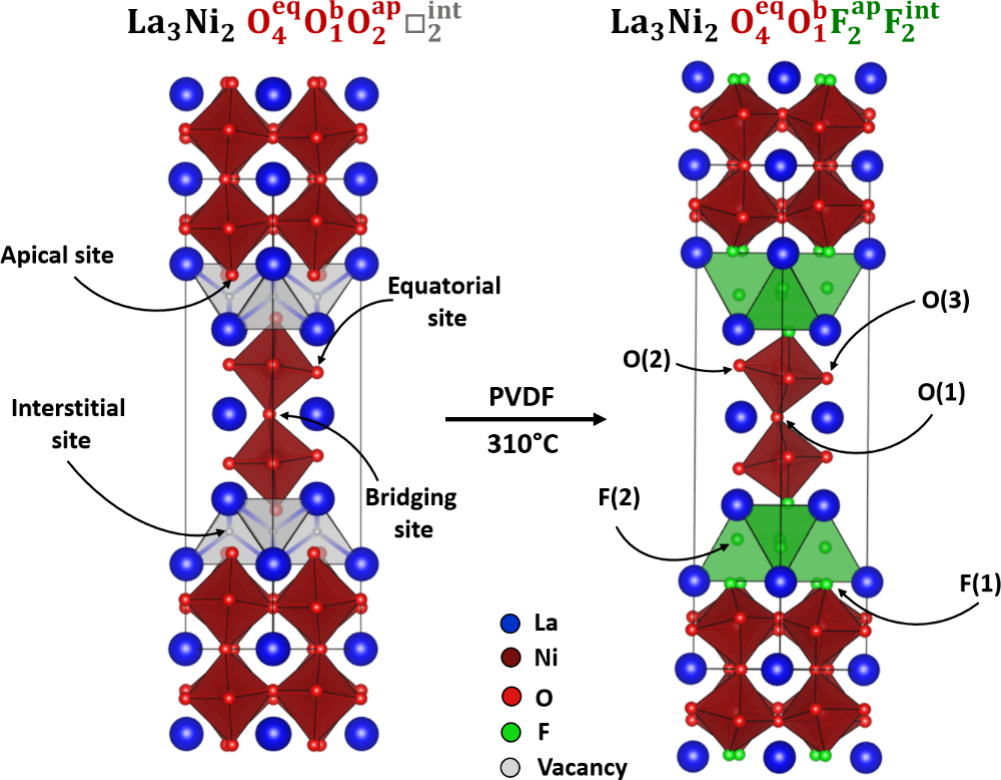
Conversion of La_3_Ni_2_O_7_ to La_3_Ni_2_O_5_F_4_.

### Structural and Chemical Characterization of La_3_Ni_2_O_5_F_3_

Heating La_3_Ni_2_O_5_F_4_ with excess LiH in an evacuated ampule at 190 °C, as described above, yielded a single-phase product. SXRD data from this material could be indexed using an orthorhombic cell (*a* = 5.639 Å, *b* = 5.649 Å, *c* = 21.15 Å). It can be seen that on reaction with LiH the *c*-parameter has shrunk by about 1.3 Å to reach an intermediate value between that of La_3_Ni_2_O_7_ and La_3_Ni_2_O_5_F_4_ indicating deintercalation of anions. Neutron powder diffraction data collected from this material at room temperature could be indexed using the same orthorhombic cell. A series of structural models were constructed based on a symmetry analysis of distorted *n* = 2 Ruddlesden–Popper phases,^23^ and refined simultaneously against the SXRD and NPD data as described in the Supporting Information. Diffraction reflections indicating the presence of LiF were also observed so this was added as a secondary phase. The best fit was achieved using a model described in space group *Pbcm* (#57). Describing the structure in a cell with *Pbcm* symmetry results in the interstitial anion site being split into two 4*c* sites which form stripes parallel to the *y*-axis. Refinement of the anion site occupancies led to the occupancy of one of the 4*c* interstitial anion sites dropping to zero, with all other anion site occupancies remaining at 1, resulting in a stripe-ordered occupation of the interstitial anion sites. Refined crystallographic parameters are given in Table 1, selected bond lengths in Table S4, plots of the data are given in Figures 2, S8 and S9 and the corresponding crystal structure is pictured in Figures 3 and S10.

**Figure 2:**
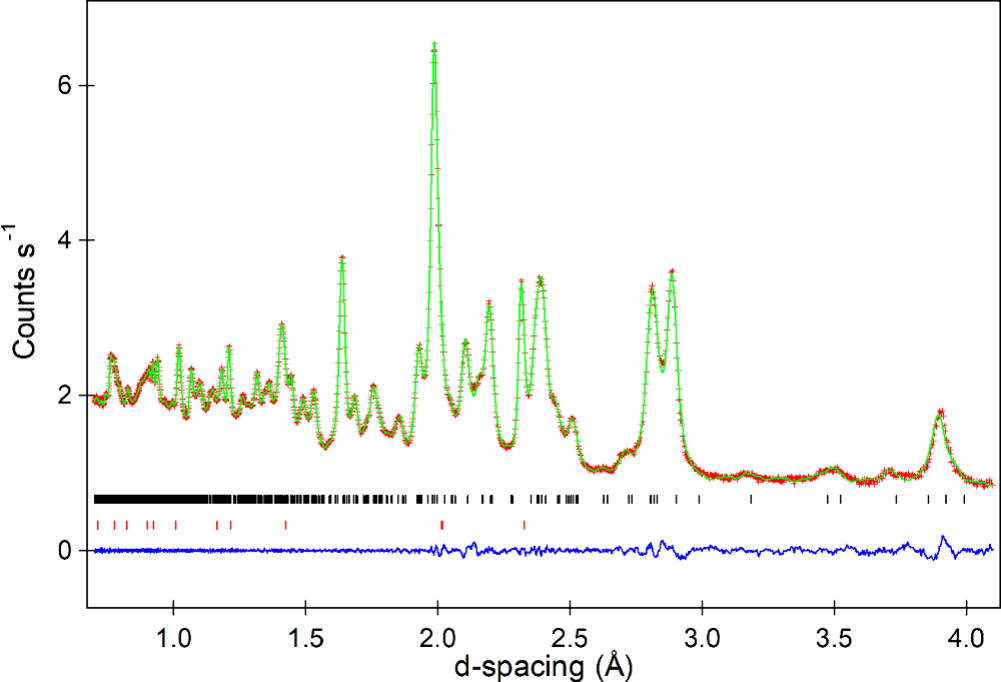
Observed, calculated and difference plots from the structural refinement of La_3_Ni_2_O_5_F_3_ against neutron powder diffraction data. Tick marks denote Bragg positions for the majority phase (black) and LiF (red).

**Table 1:** Crystallographic Parameters Extracted from the Structural Refinement of La_3_Ni_2_O_5_F_3_ against NPD Data

Atom	*x*	*y*	*z*	Occ.	B_iso_ (Å^2^)
La(1)	0.218(3)	0.524(1)	1/4	1	0.85(4)
La(2)	0.256(1)	0.493(1)	0.073(1)	1	0.85(4)
Ni(1)	0.251(1)	0.021(1)	0.158(1)	1	0.51(6)
O(1)	0.326(2)	0.041(3)	1/4	1	1.19(5)
O(2)	0.540(2)	0.238(2)	0.136(1)	1	1.19(5)
O(3)	0.062(2)	0.301(2)	0.167(1)	1	1.19(5)
F(1)	0.173(1)	0.021(1)	0.048(1)	1	1.03(9)
F(2)	0.539(1)	1/4	0	1	1.03(9)
La_3_Ni_2_O_5_F_3_, space group *Pbcm* (# 57); *a* = 5.6385(5) Å, *b* = 5.652(1) Å, *c* = 21.148(2) Å; *V* = 673.6(2) Å^3^; Formula weight = 671.11 g·mol^–1^, *Z* = 4; Weight fraction: 97.5%					
LiF, space group *Fm3̅m* (#225); *a* = 4.0294(2) Å, *V* = 65.40(6); Weight fraction: 2.5%					
Radiation source: time-of-flight neutron; Temperature: 300 K; *R*_*wp*_ = 3.18%, *R*_*p*_ *=*2.25%					

**Figure 3:**
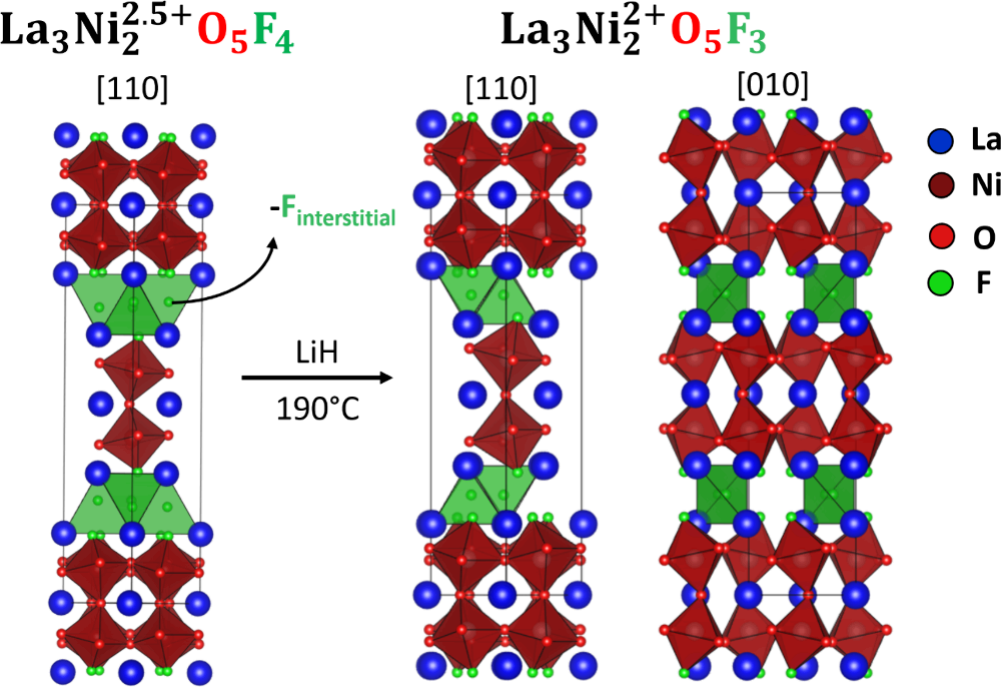
Topochemical reduction of La_3_Ni_2_O_5_F_4_ at 190 °C yields La_3_Ni_2_O_5_F_3_ with a stripe ordering of fluorine within the rock salt layer.

The NPD data unambiguously confirm the reduced compound has 8 anions per formula unit. However, the small neutron scattering contrast between O and F prevents direct determination of the anion composition and distribution of the phase from these data. Ni K-edge X-ray absorption spectroscopy data collected from the reduced phase (Figure 4) almost perfectly match those of the Ni^2+^ standard, La_2_NiO_4_, and agree with iodometric titration data (titrated oxidation state: Ni^2.03+^) allowing an anion composition of La_3_Ni_2_O_5_F_3_ for the phase to be calculated by charge balancing. This composition was confirmed via TGA data (Figure S11) Bond valence sums calculated for all anion sites lead us to conclude that the anion ordering from the parent compound was conserved as shown in Figure 3 and described in Table S5.

**Figure 4:**
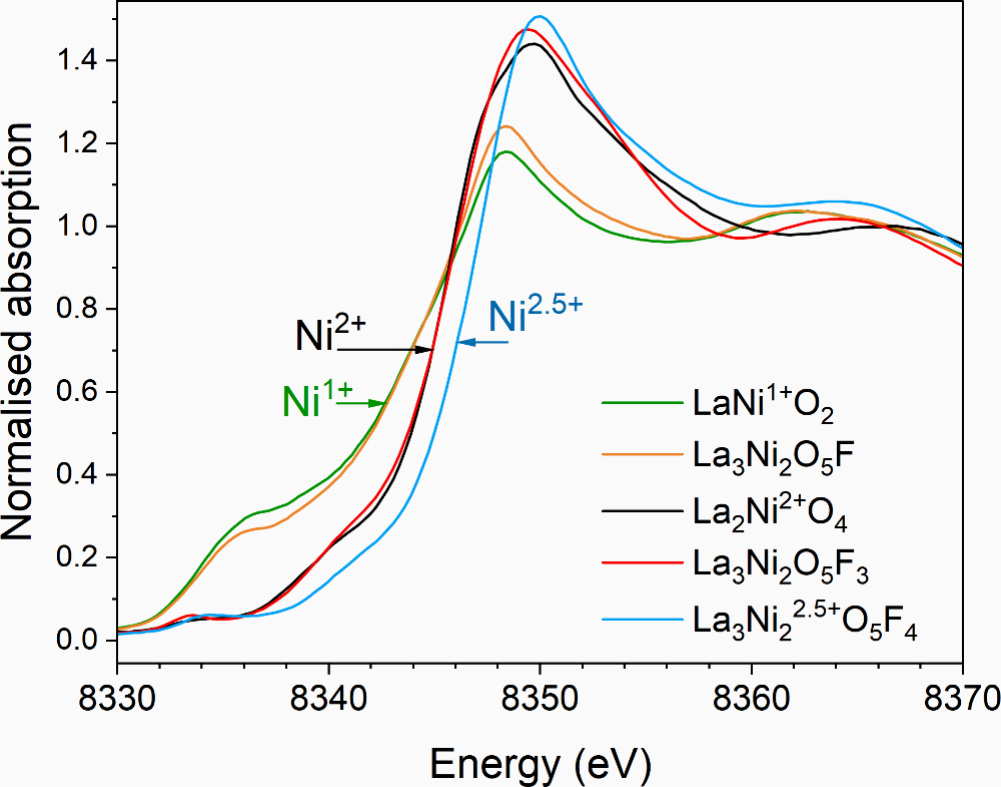
Ni K-edge XANES spectra of La_3_Ni_2_O_5_F_3_ and La_3_Ni_2_O_5_F. The spectra are referenced against LaNi^1+^O_2_, La_2_Ni^2+^O_4_ and La_3_Ni_2_O_5_F_4_.

### Structural Characterization of La_3_Ni_2_O_5_F

Reduction of La_3_Ni_2_O_5_F_4_ with LiH at 250 °C yielded a phase with a diffraction pattern which could be readily indexed with a tetragonal unit cell (*a* = 3.984 Å, *c* = 19.297 Å) together with diffraction features from LiF and LaOF impurities arising from F ion deintercalation and partial decomposition of the sample, respectively. These lattice parameters are broadly consistent with a T’ structure as could be expected for a low valent nickel compound adopting square planar coordination environments. Thus, a model based on the crystal structure of La_3_Ni_2_O_6_ (*I*4/*mmm*, #139)^13^ was made and refined simultaneously against the SXRD and NPD data (with LiF and LaOF added as secondary phases) to give good fits to both data sets (Figures 5, S12 and S13). The refined model indicates that the phase prepared via reduction at 250 °C is isostructural with La_3_Ni_2_O_6_ and has fully occupied equatorial and interstitial anion sites. As a result, the structure consists of double infinite sheets of corner-linked NiO_4_ squares separated by La­(O/F)_2_ fluorite sheets, as pictured in Figure 6. Full details of the crystallographic parameters are given in Table 2 and selected bond lengths in Table S6.

**Figure 5:**
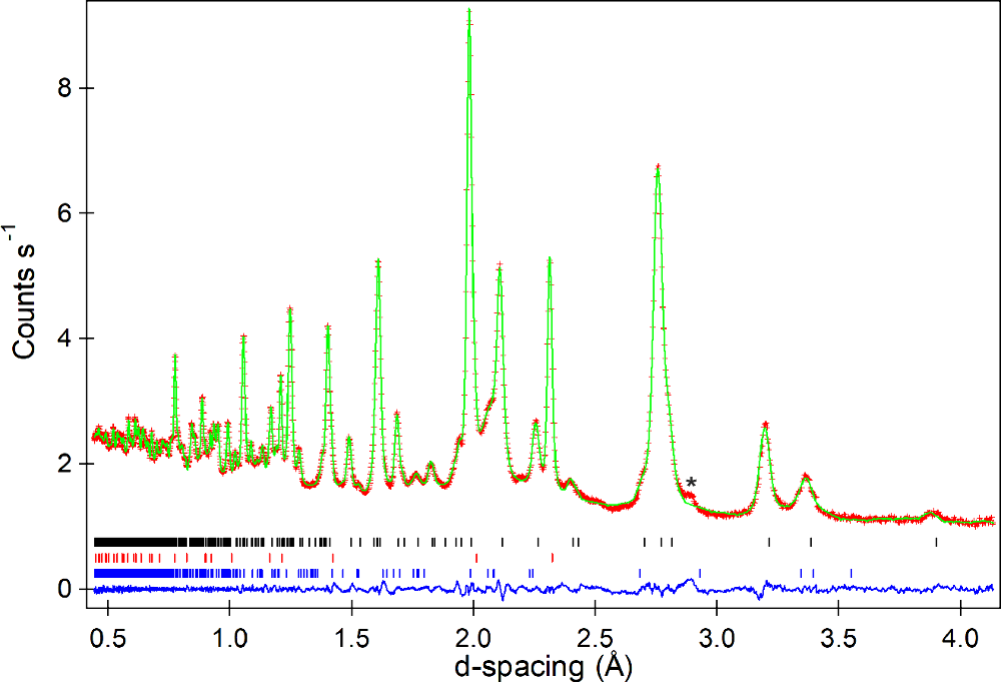
Observed, calculated and difference plots from the structural refinement of La_3_Ni_2_O_5_F against neutron powder diffraction data. Tick marks denote Bragg positions for the majority phase (black), LiF (red) and LaOF (blue). Unindexed peak at *d* ≈ 2.9 Å is an unidentified LaO_*x*_F_*y*_ secondary phase.

**Table 2:** Crystallographic Parameters Extracted from the Structural Refinement of La_3_Ni_2_O_5_F against NPD Data

Atom	*x*	*y*	*z*	Occ.	B_iso_ (Å^2^)
La(1)	0	0	1/2	1	0.22(3)
La(2)	0	0	0.3212(2)	1	0.20(2)
Ni(1)	0	0	0.0843(2)	1	0.48(2)
O(1)	0	1/2	0.0867(2)	1	0.20(2)
O/F(2)	0	1/2	1/4	0.5/0.5	0.30(4)
La_3_Ni_2_O_5_F, space group *I*4/*mmm* (# 139); *a* = 3.9838(2) Å, *c* = 19.295(2) Å, *V* = 305.94(2) Å^3^; Formula weight = 633.11 g·mol^–1^, *Z* = 2; Weight fraction: 84.7%					
LiF, space group *Fm3̅m* (#225); *a* = 4.0263(1) Å, *V* = 65.273(6) A^3^; Weight fraction: 3.9%					
LaOF, space group *R*-3*m* (#166); *a* = 4.153(1) Å, *c* = 20.112(1) Å, *V* = 300.4(3) A^3^; Weight fraction: 11.4%					
Radiation source: time-of-flight neutron; Temperature: 300 K; R_*wp*_ = 2.66%, R_*p*_ *=* 1.82%					

**Figure 6:**
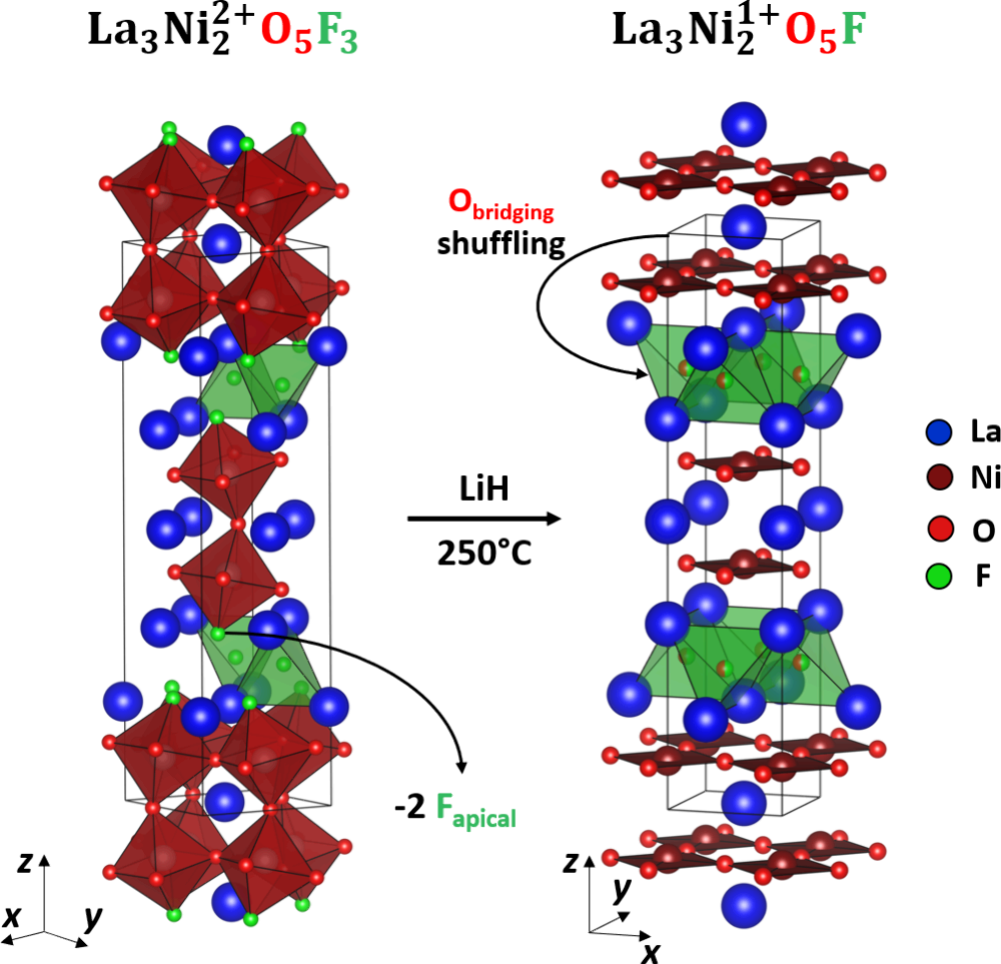
Topochemical reduction of La_3_Ni_2_O_5_F_3_ at 250 °C yields La_3_Ni_2_O_5_F, a T′ *n* = 2 Ruddlesden–Popper phase with a mixed O/F occupancy of the interstitial site.

Again, the anion composition of the reduced phase was determined using the charge balance method by observing that the XANES spectrum from the phase prepared at 250 °C matches well with that of LaNiO_2_ (Figure 4). It should be noted that the broad but intense absorption feature at 8335 eV in the data from both La_3_Ni_2_O_5_F and LaNiO_2_ is not a pre-edge transition but instead arises from the square planar coordination of Ni which lifts the degeneracy of the *t*_*1u*_ states (4p_*z*_, 4p_*y*_, 4p_*z*_ *)* such that the orbital perpendicular to the plane is stabilized. This composition was confirmed via TGA data (Figure S15).

### Magnetic Characterization

Zero-field cooled (ZFC) and field cooled (FC) magnetization data collected from La_3_Ni_2_O_5_F_4_ in an applied field of 100 Oe can be fit by a Curie–Weiss law in the range 230 K < *T* < 350 K to yield parameters C = 1.84(3) cm^3^·K·mol^–1^ and θ = −484(12) K (Figure S16). This Curie constant corresponds to a magnetic moment of μ_eff_ = 2.71 μ_B_ per Ni, slightly larger than the value of 2.34 μ_B_ per Ni calculated for a 1:1 mixture of high-spin Ni^2+^ and low-spin Ni^3+^ using the spin-only formula. The large Weiss constant indicates strong magnetic interactions over the whole temperature range. Below 215 K, the ZFC and FC data diverge weakly, consistent with a magnetic ordering transition. A magnetization-field isotherm collected at 5 K (Figure S17) exhibits slight hysteresis and does not saturate up to 5 T suggesting an antiferromagnetic (AFM) state at low temperature.

Similar magnetic behavior is observed for La_3_Ni_2_O_5_F_3_, with ZFC and FC data diverging around 225 K (Figure 7). However, above this temperature, the magnetization data are essentially temperature-independent and cannot be fit by the Curie–Weiss law. Magnetization-field data collected at 5 K, 200 and 300 K, after cooling in 5 T from 300 K, have a sigmoidal shape consistent with the presence of a small amount of Ni metal impurity (Figure S18). Again, no saturation is observed up to 5 T and the moment is small, suggesting AFM ordering. Data collected at 5K and 200 K do not exhibit hysteresis, while the 5 K data exhibit weak hysteresis and are displaced from the origin, consistent with a glassy component to the magnetic order.

**Figure 7:**
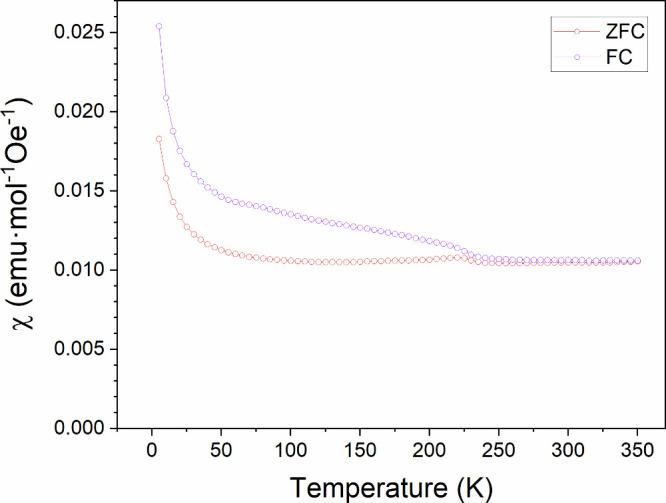
Field cooled and zero-field cooled magnetic susceptibility data collected from La_3_Ni_2_O_5_F_3_ as a function of temperature in an applied field of 100 Oe.

Neutron powder diffraction data collected from La_3_Ni_2_O_5_F_3_ at 10 K show a series of additional peaks not observed in the analogous data collected at room temperature, indicative of magnetic ordering (Figure 8). The additional reflections could be indexed using the crystallographic unit cell and the diffraction features from the nuclear structure could still be fit by the room temperature structural model described in space group *Pbcm*, suggesting a magnetic propagation vector **k** = (0, 0, 0). A series of magnetic structural models were constructed on this basis using the ISODISTORT^24^ software package, and these models were refined against the NPD data. The best fit to the data was achieved by applying the mΓ_2_^+^ magnetic irreducible to the *Pbcm* crystallographic structure to yield a model described in the magnetic space group *Pb’c’m* (#57.382), as shown in Figure S21. This magnetic structure consists of a G-type antiferromagnetic ordering of the nickel moment aligned along the *x*-axis with a magnitude of 1.57(1) μ_B_ per Ni center as shown in Figure 8. The *Pb’c’m* space group allows a ferromagnetic alignment of spins parallel to the *z*-axis, but this contribution declined to zero when refined.

**Figure 8:**
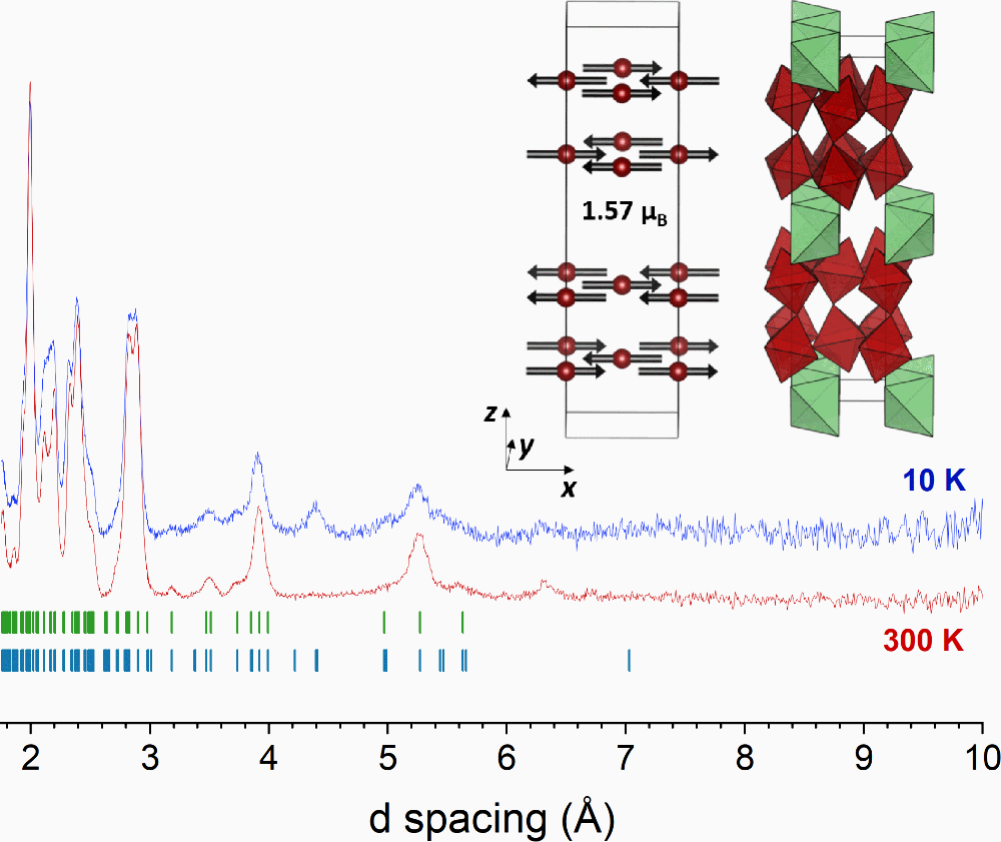
Comparison of the NPD data collected from La_3_Ni_2_O_5_F_3_ (bank 2 of the POLARIS diffractometer) at 300 and 10 K. Green ticks correspond to nuclear Bragg peaks and blue ticks correspond to magnetic peaks. The magnetic structure of G-type antiferromagnetically ordered La_3_Ni_2_O_5_F_3_ is also represented. NiO_5_F octahedra are represented in dark red and FLa_4_ tetrahedra in light green.

In contrast to the other La–Ni–O-F phases, magnetization data collected from La_3_Ni_2_O_5_F in an applied field of 100 Oe do not show any divergence between ZFC and FC data and do not obey the mathematical form of the Curie–Weiss law (Figure S19). Magnetization-field data indicate this sample contains a significant amount of elemental Ni unseen in the diffraction data (Figure S20), therefore a ‘ferrosubtraction’ measurement was carried out, as described in detail in the SI, which allowed the separation of the ferromagnetic and paramagnetic components of the data as shown in Figure 9. These data show a broad maximum in the paramagnetic susceptibility of La_3_Ni_2_O_5_F at 95 K.

**Figure 9:**
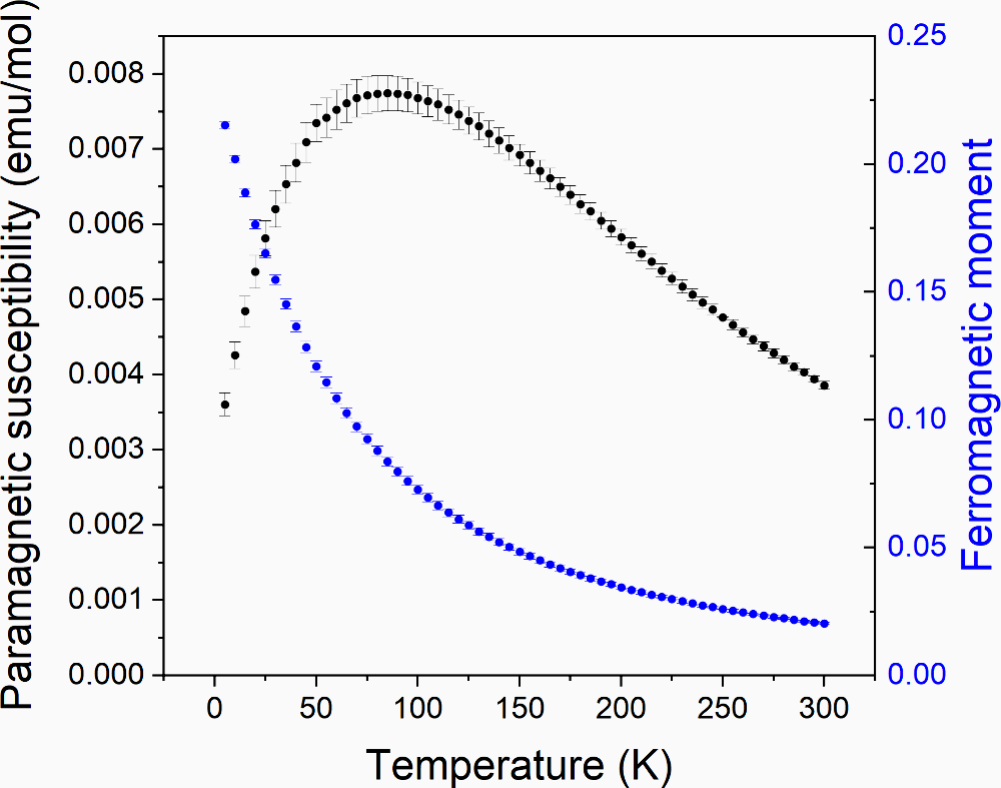
Plots of paramagnetic susceptibility and saturated ferromagnetic moment, as a function of temperature, for La_3_Ni_2_O_5_F as determined by the “ferrosubtraction method”.

## Discussion

The fluorination of La_3_Ni_2_O_7_ with PVDF yields La_3_Ni_2_O_5_F_4_. In contrast, fluorination of La_3_Ni_2_O_7_ with CuF_2_ under oxygen, yields La_3_Ni_2_O_5.5_F_3.5_.^22^ The differing products resulting from the two fluorination procedures can be rationalized by considering the redox conditions during the fluorinations. Heating CuF_2_ under oxygen yields an oxidizing F_2_/O_2_ mixture. However, the principal fluorine containing species produced by the decomposition of PVDF is HF,^25,26^ which is accompanied by a variety of carbon containing species which act to lower the partial pressure of oxygen in the system, so the conditions, when PVDF is the fluorinating agent are less oxidizing than when CuF_2_/O_2_ is the fluorinating agent. As a consequence, fluorination with PVDF results in La_3_Ni_2_O_5_F_4_, a phase with the same average nickel oxidation state as La_3_Ni_2_O_7_ (Ni^2.5+^), while fluorination with CuF_2_/O_2_ yields La_3_Ni_2_O_5.5_F_3.5_ with an average oxidation sate of Ni^2.75+^ which is oxidized compared to the all-oxide starting phase, consistent with the more oxidizing reaction conditions.

Reduction of La_3_Ni_2_O_5_F_4_ with LiH proceeds via a series of anion extraction reactions. In the first step a single fluoride ion per formula unit is deintercalated to form the Ni^2+^ phase La_3_Ni_2_O_5_F_3_.

In principle an equivalent change in oxidation state could have occurred via the deintercalation of half an oxide ion per formula unit. However, as noted previously,^15^ fluorinated Ruddlesden–Popper phases tend to have short distances between their interstitial and apical anion sites (2.58 – 2.77 Å in this instance) so removal of fluoride ions from either the apical or interstitial sites of La_3_Ni_2_O_5_F_4_, (rather than oxide ions from the equatorial or bridging anion sites) will relieve some of the anion–anion repulsion in the system, and thus appears to be favored. The observed preference to initially remove anions from the interstitial sites of La_3_Ni_2_O_5_F_4_ on reduction, rather than the apical sites (the preference observed during the analogous reduction of LaSr_2_CoRuO_5.5_F_3.5_)^15^ can be rationalized by noting that Ni^2+^ has a strong preference for octahedral coordination, and this coordination is conserved if interstitial rather than apical anions are extracted. Thus, removal of half the interstitial fluoride ions relieves some of the anion–anion repulsion in the system while maintaining an octahedral geometry around the nickel cations.

The fluoride ions within the half-filled interstitial layers of La_3_Ni_2_O_5_F_3_ order into stripes parallel to the *y*-axis, as shown in Figure 3. This anion ordering is accompanied by a change in the tilt scheme of the NiX_6_ octahedra, from *a* ^*–*^ *a* ^*–*^ *c* ^*+*^ */a* ^*–*^ *a* ^*–*^ *-c* ^*+*^ in La_3_Ni_2_O_5_F_4_ to *a* ^*–*^ *a* ^*–*^ *c* ^*+*^ */-(a* ^*–*^ *a* ^*–*^ *)­c* ^*+*^ in La_3_Ni_2_O_5_F_3_, which further extends the separation between the apical and interstitial anion site,. This anion ordering is analogous to that seen in *n* = 1 Ruddlesden–Popper oxyfluorides La_2_NiO_3_F_2_ and La_2_CuO_3_F_2_, although in these examples the interstitial anion is oxide, rather than a mixture of oxide and fluoride.^27,28^

Magnetization and low-temperature NPD data indicate La_3_Ni_2_O_5_F_3_ adopts an antiferromagnetically ordered state below *T*_N_ ∼ 225 K. This ordering temperature is almost identical to that of the *n* = 1 Ruddlesden–Popper phase, La_2_NiO_2.5_F_3_,^29^ consistent with the related crystal structures, and common Ni^2+^ oxidation states of the two phases.

Raising the reaction temperature to 250 °C leads to the reductive extraction of two further fluoride ions and the formation of the Ni^I^ phase La_2_Ni_2_O_5_F. This reaction step involves a large-scale reorganization of the anion lattice, the net result of which is the relocation of the oxide ions in the bridging anion site to the interstitial anion site, as shown in Figure 6. While such a large-scale reorganization may appear counterintuitive, it is not unprecedented, with the transformation of Sr_2_Fe_2_O_5_ to SrFeO_2_ exhibiting a similar large-scale anion-lattice rearrangement.^30,31^

The fluorinate-then-reduce synthesis route, which first performs a redox-neutral fluorine insertion/exchange to convert La_3_Ni_2_O_7_ to La_3_Ni_2_O_5_F_4_, before reducing this to La_3_Ni_2_O_5_F_,_ allows the preparation of the Ni^I^ phase, in contrast to direct reduction of La_3_Ni_2_O_7_ which forms the Ni^1.5+^ phase La_3_Ni_2_O_6_. As a result, La_3_Ni_2_O_5_F can be factorized as (LaNiO_2_)_2_(LaOF) rather than [(LaNiO_2_)_2_]^+^[LaO_2_]^−^ as is the case for La_3_Ni_2_O_6_, emphasizing the structural and electronic similarity of the oxyfluoride and LaNiO_2_. Furthermore, this factorization explicitly shows that the O/F anion substitution that occurs on changing from La_3_Ni_2_O_6_ to La_3_Ni_2_O_5_F occurs outside the primary coordination sphere of the nickel cations, preserving the NiO_2_ infinite sheets, and thus can be considered as a “pure” electron doping of the system.

Magnetization data collected from La_3_Ni_2_O_5_F in an applied field of 100 Oe (Figure S19) are dominated by the presence of small amounts of ferromagnetic, elemental nickel in the sample. However, these data show no indication of superconductivity down to 5 K. Magnetization data collected in large applied magnetic fields (3–5 T) to eliminate the contributions from elemental nickel (Figure 9) reveal a broad maximum in the susceptibility of La_3_Ni_2_O_5_F, centered at around 75 K, suggestive of antiferromagnetic order.

## Conclusions

By sequentially applying multiple topochemical reactions to a host, novel materials can be synthesized, which are not attainable by other routes. Thus, by fluorinating La_3_Ni_2_O_7_ to La_3_Ni_2_O_5_F_4_ prior to reduction, the Ni^1+^ phase La_3_Ni_2_O_5_F can be prepared, rather than the product of direct reduction, the Ni^+1.5^ phase La_3_Ni_2_O_6_. The net effect of using the fluorinate-then-reduce procedure, rather than direct reduction, is to electron-dope the double infinite-layer phase La_3_Ni_2_O_6_ via fluoride-for-oxide anion substitution. This suggests that by applying this reaction sequence to a partially oxidized *A*_3_Ni_2_O_7_ phase, a reduced, double infinite-layer material with the optimum doping level for superconductivity (Ni^∼+1.2^) could be synthesized.

## Supplementary Material



## Notes

Details of sample preparation, additional NPD and SXRD data refinements, bond valence sums calculations, TGA plots and additional magnetic measurements (PDF)
