# Supplementary material for: Synthesis of the Double Infinite-Layer Ni(I) Phase La3Ni2O5F via Sequential Topochemical Reactions
Source: J Am Chem Soc. 2026 Feb 4;148(6):6109–15. doi: 10.1021/jacs.5c16740 (PMC12921833; doi:10.1021/jacs.5c16740)
Supplement: Supplementary file 1 [file ja5c16740_si_001.pdf]

Synthesis of the Double Infinite-Layer Ni(I) phase $La_3Ni_2O_5F$ via Sequential Topochemical Reactions.

Romain Wernert, Robert D. Smyth and Michael A. Hayward*

Supporting Information

Table of Contents

1. Structural Characterisation of $La_3Ni_2O_7$
Figure S1. Observed calculated and difference plots from the structural refinement of $La_3Ni_2O_7$ against SXRD data collected at room temperature.
Figure S2. Crystal structure of the n = 2 Ruddlesden-Popper aristotype (*I*4/*mmm*) with the labelling of the anion sites as referred to in this manuscript.

2. Sample Preparation

3. Characterisation of $La_3Ni_2O_5F_4$
Figure S3. Observed calculated and difference plots from the structural refinement of $La_3Ni_2O_5F_4$ against SXRD data.
Figure S4. Crystal structure of $La_3Ni_2O_5F_4$ viewed down the [110] direction.
Table S1: Crystallographic parameters extracted from the structural refinement of $La_3Ni_2O_5F_4$ against SXRD data.
Table S2. Selected bond lengths from the refined structure of $La_3Ni_2O_5F_4$.
Figure S5. X-ray absorption near edge spectra (XANES) collected at the Ni K-edge for $La_3Ni_2O_5F_4$. The spectrum is referenced against $La_2Ni^{2+}O_4$, $La_3Ni_2^{2.5+}O_7$ and $LaNi^{3+}O_3$.
Figure S6. Observed, calculated and difference plots from the fit to the XRPD data collected from a sample of $La_3Ni_2O_5F_4$ annealed at 1000 °C.
Figure S7. Thermogravimetric data collected while heating $La_3Ni_2O_5F_4$ under a 10:90 H_2:N_2 gas flow.
Table S3. Results of the bond valence sum calculations of the different anion sites in $La_3Ni_2O_5F_4$.

4. Characterisation of $La_3Ni_2O_5F_3$
Figure S8: Observed, calculated and difference plots from the structural refinement of a *Pnam* symmetry model against SXRD data collected from $La_3Ni_2O_5F_3$.
Figure S9. Observed, calculated and difference plots from the structural refinement of a *Pbcm* symmetry model against NPD data collected from $La_3Ni_2O_5F_3$ using the 5 detector banks of the POLARIS instrument at room temperature.
Table S4. Selected bond lengths from the refined structure of $La_3Ni_2O_5F_3$.
Figure S10. Crystal structure of $La_3Ni_2O_5F_3$ viewed down the [110] direction.
Figure S11. Thermogravimetric data collected while heating $La_3Ni_2O_5F_3$ under a 10:90 H_2:N_2 gas flow.
Table S5. Results of the bond valence sum calculations of the different anion sites in $La_3Ni_2O_5F_3$.

5. Characterisation of $La_3Ni_2O_5F$
Figure S12. Observed, calculated and difference plots from the structural refinement of a *I*4/*mmm* symmetry model against SXRD data collected from $La_3Ni_2O_5F$.
Figure S13: Observed, calculated and difference plots from the structural refinement of a *I*4/*mmm* symmetry model against NPD data collected from $La_3Ni_2O_5F$ using the 5 detector banks of the POLARIS instrument at room temperature.
Table S6. Selected bond lengths from the refined structure of $La_3Ni_2O_5F$.
Figure S14. Crystal structure of $La_3Ni_2O_5F$.
Figure S15. Thermogravimetric data collected while heating $La_3Ni_2O_5F$ under a 10:90 H_2:N_2 gas flow.

6. Magnetic characterisation
Procedure used to measure the magnetization of samples containing elemental nickel.
Figure S16. Field cooled and zero field cooled magnetisation data collected from $La_3Ni_2O_5F_4$ as a function of temperature in an applied field of 100 Oe (left) and corresponding reciprocal curve fitted to the Curie-Weiss law (right).
Figure S17. Magnetization data collected from $La_3Ni_2O_5F_4$ at 300 K and 5 K, as a function of applied field. The data at 5 K were collected after field cooling from 300 K in an applied field of 50 000 Oe.

Figure S18. Magnetization data collected from $La_3Ni_2O_5F_3$ at 300 K, 200 K and 5 K, as a function of applied field. The data at 5 K and 200 K were collected after field cooling from 300 K in an applied field of 50 000 Oe.

Figure S19. Field cooled and zero field cooled magnetisation data collected from $La_3Ni_2O_5F$ as a function of temperature in an applied field of 100 Oe.

Figure S20. Magnetization data collected from $La_3Ni_2O_5F$ at 300 K and 5 K, as a function of applied field. The data were collected at 5 K after field cooling from 300 K in an applied field of 50 000 Oe.

Figure S21. Observed, calculated and difference plots from the structural refinement of a *Pb'c'm* symmetry model against NPD data collected from $La_3Ni_2O_5F_3$ at 10 K.

Figure S22. Magnetization of $La_3Ni_2O_5F$ measured as a function of applied field at 300 K.

1. Structural Characterisation of $La_3Ni_2O_7$

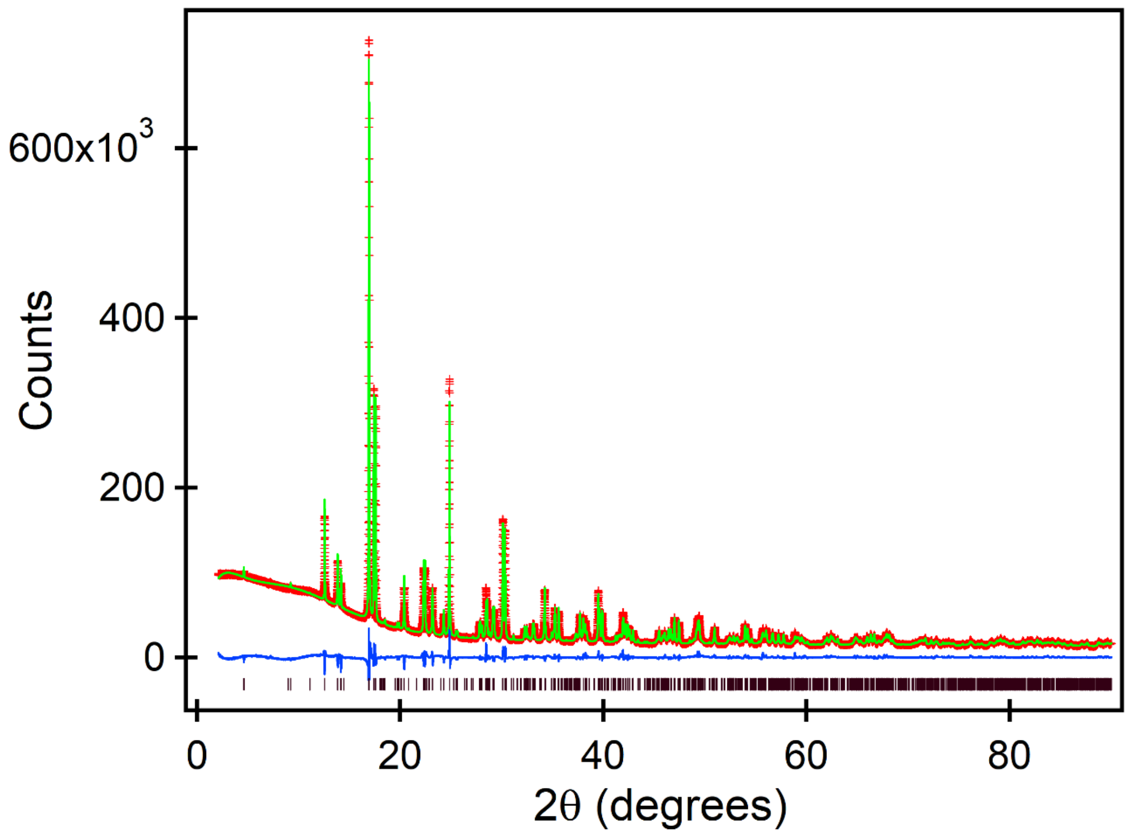

Figure S1. Observed calculated and difference plots from the structural refinement of $La_3Ni_2O_7$ against SXRD data collected at room temperature. Refined lattice parameters: *a* = 5.3914(2) Å, *b* = 5.4467(2) Å, *c* = 20.5186(6) Å, space group *Amam* (#63).

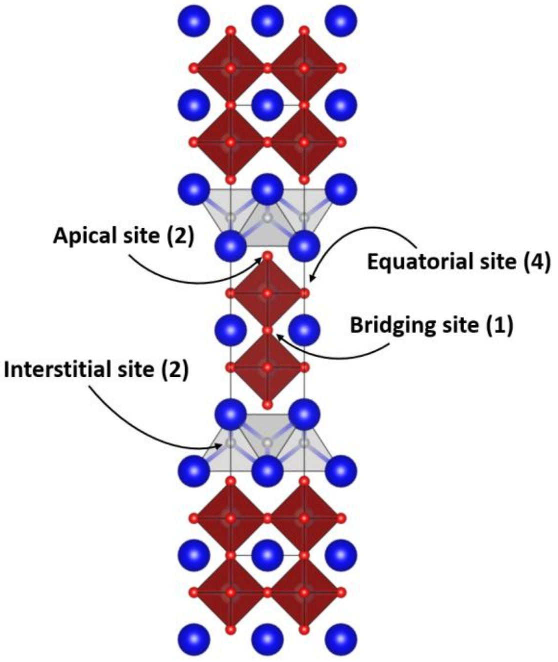

Figure S2. Crystal structure of the n = 2 Ruddlesden-Popper aristotype (*I*4/*mmm*) with the labelling of the anion sites as referred to in this manuscript. The multiplicity of anion sites per formula unit is given in the brackets.

2. Standard Sample Preparation

A 1-gram sample of La_2NiO_4 was made by grinding a suitable stoichiometric ratio of NiO (99.99%) and La_2O_3 (99.999%, dried overnight at 900°C) in a mortar and pestle. The powder was pressed into a 13 mm diameter pellet and heated to 1000 °C under Ar for two cycles of 12 hours with intermediate regrinding followed by annealing at 350°C under 5% H_2 in N_2. The lab XRD pattern was refined in the *Bmab* space group with lattice parameters *a* = 5.4666(2) Å, *b* = 5.5384(2) Å and *c* = 12.5435(2) Å, which match those reported by Jorgensen *et al.* (*a* = 5.4656 Å, *b* = 5.5327 Å and *c* = 12.5547 Å).[1]

A 1-gram sample of $LaNiO_3$ was made by dissolving a suitable stoichiometric ratio of Ni (99.99%) and La_2O_3 (99.999%, dried overnight at 900°C) in 50 mL deionized water to which a minimum amount of nitric acid was added. The metal ions were precipitated using tetramethylammonium hydroxide (25% in H_2O) and the green precipitate was washed with water and recovered by vacuum assisted filtration. The product obtained was then heated to 500°C in air for 12 hours to burn off the remaining organic components, ground into a fine powder, pressed into a 13mm diameter pellet and heated to 850°C under air for two cycles of 12 hours with intermediate regrinding. The lab XRD pattern was refined in the *R*-3*c* space group with lattice parameters *a* = 5.4558(3) Å and *c* = 13.1643(8) Å

A 500 mg sample of $LaNiO_2$ was made by grinding $LaNiO_3$ with 4 mole equivalents of NaH (99%). The powder was sealed in an evacuated ampoule, heated to 200°C for two cycles of 48 hours each with intermediate regrinding. The product was then washed with methanol to remove the unreacted NaH and the NaOH by-product, dried and then kept in an argon-filled glovebox. The lab XRD pattern was refined in *P*4/*mmm* space group with lattice parameters *a* = 3.9607(6) Å and *c* = 3.4046(5) Å.

3. Characterisation of $La_3Ni_2O_5F_4$

SXRD data collected from the product of fluorination of $La_3Ni_2O_7$ could be indexed using an orthorhombic unit cell *a* = 5.4243(2) Å, *b* = 5.5039(2) Å, *c* =22.462(1) Å with reflection conditions consistent with space group *Pnam* (#62). A structural model based on the reported structure of $La_3Ni_2O_{5.5}F_{3.5}$ was refined against the data to achieve a good fit as shown in Figure S3. In the final refinement cycle all lattice parameters and atomic positional and displacement parameters were allowed to vary along with peak shape parameters, instrumental zero-point and background parameters. To confirm the anion stoichiometry of the phase the occupancies of all anion sites we allowed to vary, and it was observed that all refined to full occupancy within err (< 1.5%). Furthermore when the anion occupancy were set to artificially low values the fit to the data declined noticeably, for example setting the occupancy of the F(2) anion site to 0.75 raised the R_{Brag} fit to 3.18, compared to R_{Brag} = 3.06 at full occupancy. Thus, the occupancy of all the anion sites was set at unity, indicating an overall sample stoichiometry of $La_3Ni_2(O/F)_9$. Full details of the refined structural parameters are given in Table S1, with selected bond lengths in Table S2.

XANES data (Figure S5) indicate an average Ni oxidation state of Ni + 2.5, which was also confirmed by iodometric titration. We can therefore deduce that the total charge of the 9 anions is -14, indicating a stoichiometry of $La_3Ni_2O_5F_4$.

This value was confirmed by heating a portion of the sample in an evacuated silica ampoule to 1000 °C. X-ray diffraction data collected from this sample after annealing (Figure S6) can bit fitted by a model containing a 3:2 molar ratio of LaOF:NiO, consistent with reaction (1)

$$La_3Ni_2O_5F_4 \Rightarrow 3LaOF + 2\ NiO + \frac{1}{2} F_2 \quad (1)$$

In addition, thermogravimetric data collected while heating $La_3Ni_2O_5F_4$ under a 10:90 H_2:N_2 gas mixture resulted in a mass loss of 5.9% (Figure S7). X-ray diffraction data collected from the products of the process indicate sample decomposed to a mixture of LaOF + LaF_3 + Ni. Thus, a mass loss of 5.9% is consistent with an initial composition $La_3Ni_2O_{5.08(5)}F_{3.92(5)}$.

Bond valence sums calculated for the anion sites using parameters for O^{2-} and F^- (Table S3) indicate that fluoride ions occupy the apical and interstitial sites, and oxygen occupies the bridging and equatorial sites.

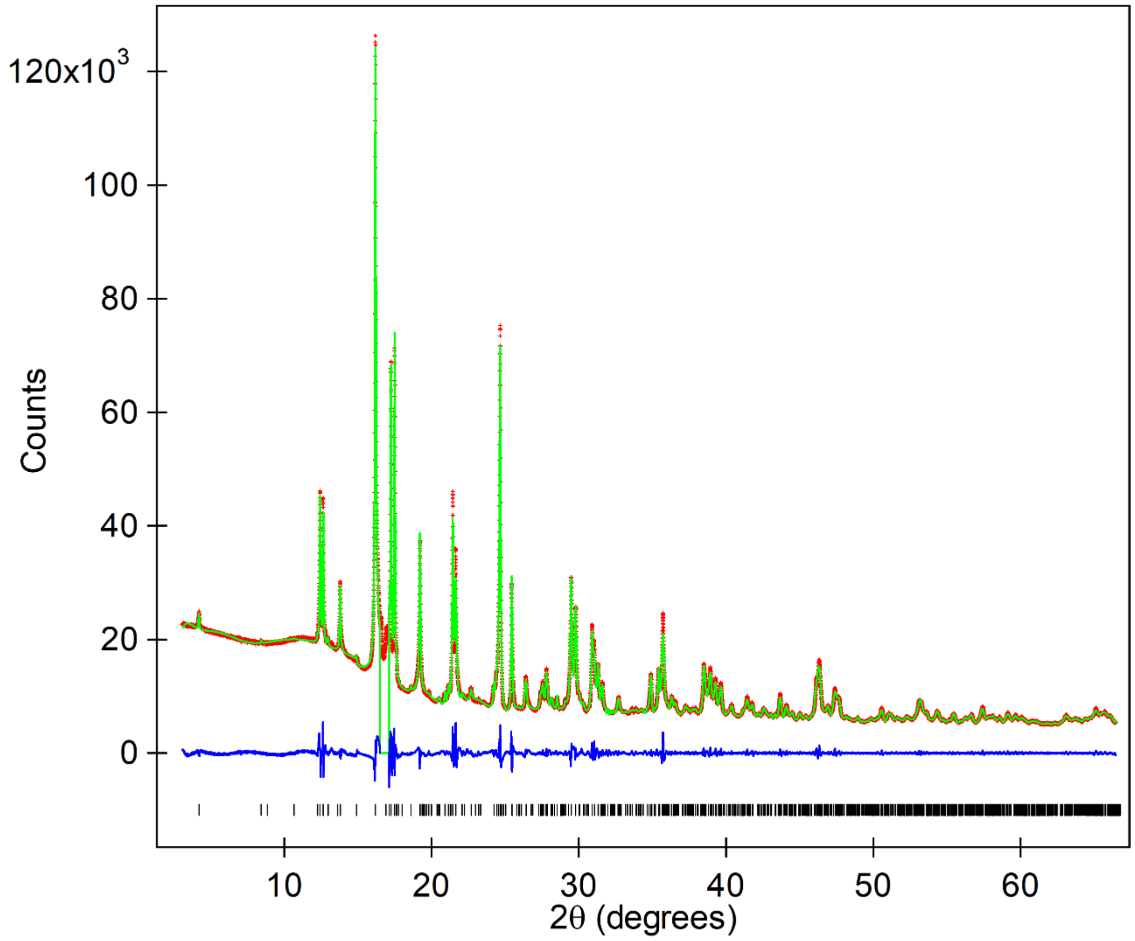

Figure S3. Observed calculated and difference plots from the structural refinement of $La_3Ni_2O_5F_4$ against SXRD data collected at room temperature. Excluded region 16.5 < 2θ < 17.1 removes a peak from an unknown secondary phase.

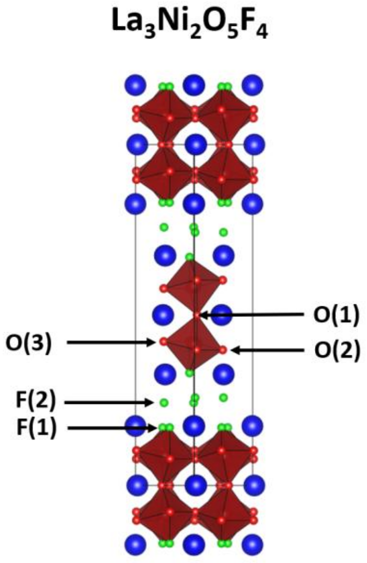

Figure S4. Crystal structure of $La_3Ni_2O_5F_4$ viewed down the [110] direction.

**Atom**	***x***	***y***	***z***	**Occupancy**	**B_{iso} (Å^2)**
La(1)	0.7224(7)	0.0014(7)	¼	1	0.5(2)
La(2)	0.7551(5)	0.9979(11)	0.0754(1)	1	0.5(2)
Ni(1)	0.7556(7)	0.0011(12)	0.8345(1)	1	0.2(2)
O(1)	0.733(1)	0.567(2)	¼	1	0.2(2)
O(2)	0.985(3)	0.276(2)	0.852(3)	1	0.2(2)
O(3)	0.450(2)	0.281(3)	0.326(2)	1	0.2(2)
F(1)	0.772(4)	0.946(4)	0.919(4)	1	0.2(2)
F(2)	0.992(5)	0.254(4)	0.993(2)	1	0.2(2)
$La_3Ni_2O_5F_4$, space group *Pnam* (# 62)<br>a = 5.4245(2) Å, b = 5.5038(2) Å, c =22.462(1) Å, V = 670.63(5) Å^3<br>Formula weight = 690.1 g·mol^{-1}, Z = 4					
Radiation source: Synchrotron X-ray radiation (λ = 0.824 Å)<br>Temperature: 300 K<br>R_{wp} = 4.86%, R_p = 2.84%, GOF = 5.87%					

Table S1. Crystallographic parameters extracted from the structural refinement of $La_3Ni_2O_5F_4$ against SXRD data.

Cation	Anion	Bond length (Å)
La(1)	O(1)	2.380(13)
	O(1)	2.678(7)
	O(1)	2.790(7)
	O(2)	2.84(6) × 2
	O(3)	2.42(4) × 2
	O(3)	2.73(4) × 2
La(2)	O(2)	2.42(5)
	O(2)	2.62(5)
	O(3)	2.74(4)
	F(1)	2.48(3)
	F(1)	2.57(3)
	F(1)	2.88(3)
	F(2)	2.48(4)
	F(2)	2.44(4)
	F(2)	2.66(4)
	F(2)	2.70(4)
Ni(1)	O(1)	1.940(4)
	O(2)	1.95(3)
	O(2)	1.99(3)
	O(3)	1.926(17)
	O(3)	2.007(15)
	F(1)	1.92(9)

Table S2. Selected bond lengths from the refined structure of $La_3Ni_2O_5F_4$.

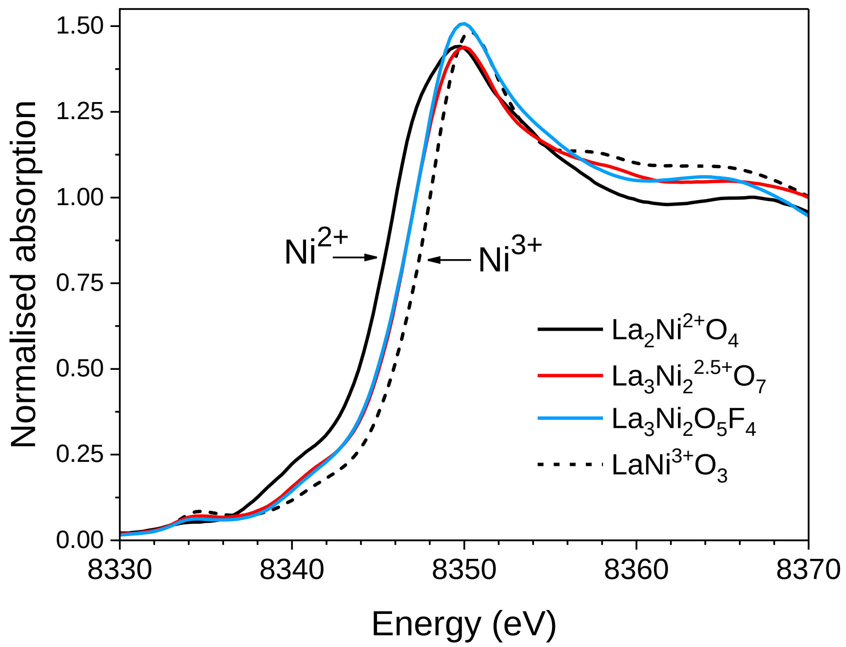

Figure S5. X-ray absorption near edge spectra (XANES) collected at the Ni K-edge for $La_3Ni_2O_5F_4$. The spectrum is referenced against $La_2Ni^{2+}O_4$, $La_3Ni_2^{2.5+}O_7$ and $LaNi^{3+}O_3$.

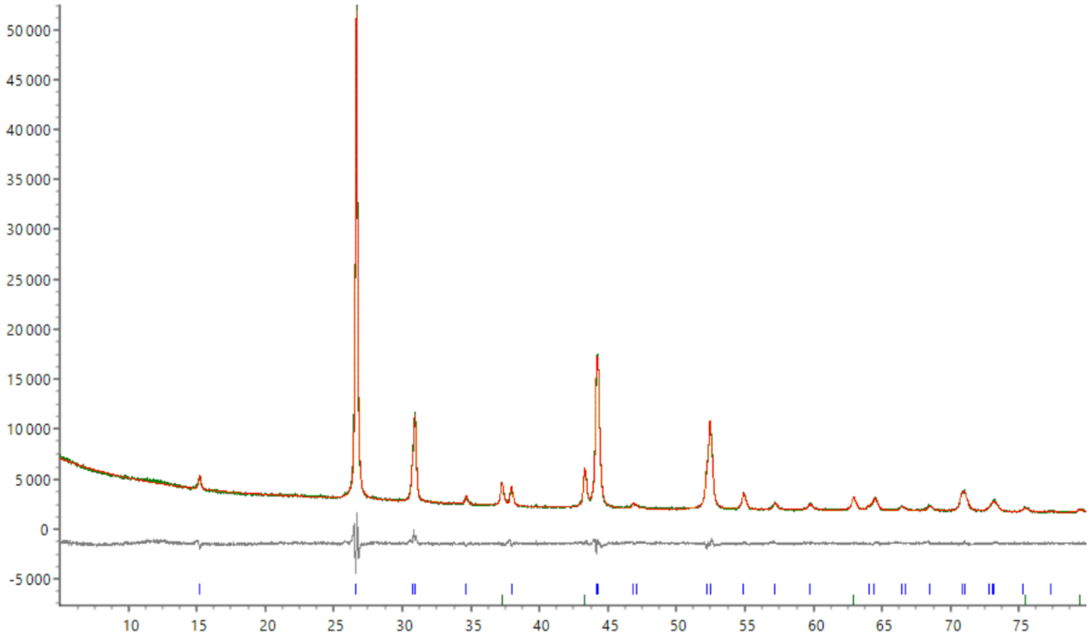

Figure S6. Observed, calculated and difference plots from the fit to the XRPD data collected from a sample of $La_3Ni_2O_5F_4$ annealed at 1000 °C in an evacuate silica tube using a model containing a 3:2 molar mixture of LaOF (blue ticks) and NiO (green ticks).

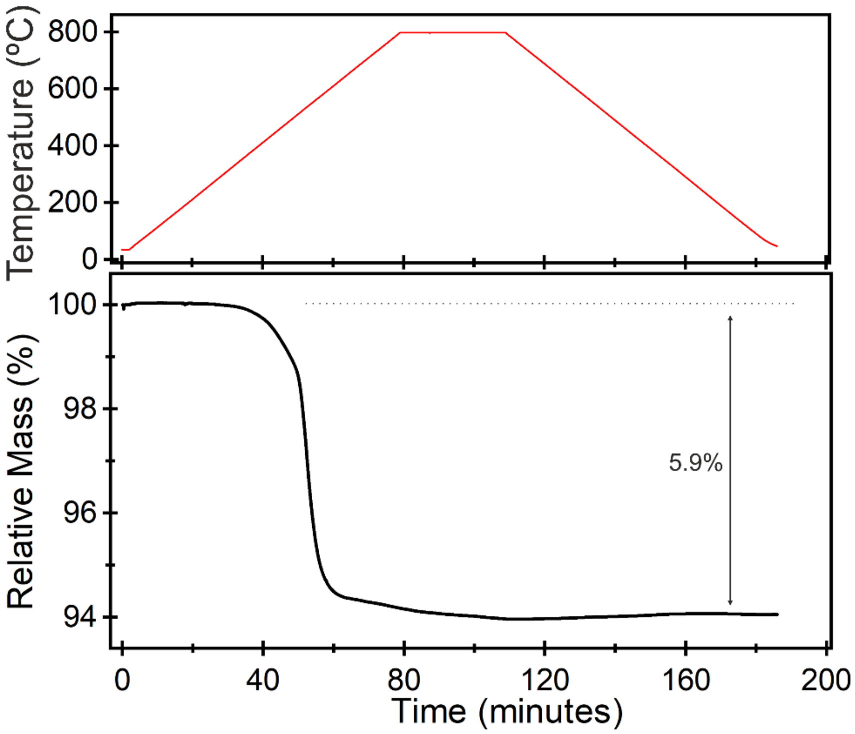

Figure S7. Thermogravimetric data collected while heating $La_3Ni_2O_5F_4$ under a 10:90 H_2:N_2 gas flow. X-ray diffraction data indicate the sample is converted to a mixture of LaOF + LaF_3 + Ni, indicating an initial composition of $La_3Ni_2O_{5.08(5)}F_{3.92(5)}$.

$La_3Ni_2O_5F_4$	BVS F	BVS O	Assignment
Apical F(1)	**1.24**	1.5	**Fluorine**
Bridging O(1)	1.66	**2.01**	**Oxygen**
Equatorial O2	1.61	**1.96**	**Oxygen**
Equatorial O3	1.5	**1.82**	**Oxygen**
Interstitial F(2)	**1.11**	1.35	**Fluorine**

Table S3. Results of the bond valence sum calculations of the different anion sites in $La_3Ni_2O_5F_4$ assuming they are occupied by either oxygen or fluorine.

4. Characterisation of $La_3Ni_2O_5F_3$

SXRD and NPD data collected from the product of reaction between $La_3Ni_2O_5F_4$ and LiH at 190 °C could be indexed using a primitive orthorhombic unit cell (*a* = 5.638 Å, *b* = 5.652 Å, *c* = 21.149 Å). A series of structural models constructed based on a symmetry analysis of distorted *n* = 2 Ruddlesden Popper phases,[23] were refined against the NPD data. Models described in space groups $P2_1nm$ (#31), *Pbcm* (#57), *P*2*cm* (#28), $P2_1/c$ (#14), *Pbcb* (#54) and *P*2/*c* (#13) were able to index the data. However, only the model described in space group *Pbcm*, in which the NiX_6 octahedra have an $a^-a^-c^+$/–(a^-a^-)c^+ ordered tilting distortion, could account for the relative intensities of the diffraction peaks with a physically reasonable configuration of atoms.

The structural model described in space group *Pbcm* has two distinct 4*c* interstitial anion sites which form stripes parallel to the *y*-axis. Refinement of the anion site occupancies led to the occupancy of one of the 4*c* interstitial anion sites dropping to zero, with all other anion site occupancies remaining at 1, resulting in a stripe-ordered occupation of the interstitial anion sites.

Weak diffraction features attributable to the presence of LiF were observe in both the SXRD and NPD data sets so this was added to the structural model as a second phase.

To accurately determine the structure of the phase, the *Pbcm* symmetry model was simultaneously refined against both the SXRD and NPD data. In the final refinement cycle all lattice parameters and atomic positional and displacement parameters were allowed to vary along with peak shape parameters, instrumental zero-point and background parameters (Cosine Fourier series) and the neutron absorption coefficients. In addition, a Stevens *hkl* dependent broadening term, which broadens reflections with a non-zero l component was also added to the model, for the majority phase. Full details for the refined structural parameters are described in Table 1 in the main text, with selected bond lengths given in Table S4 and plots of the data in Figure 2 in the main text and Figure S8 and S9.

The diffraction data indicate an overall composition of $La_3Ni_2(O/F)_8$. XANES data (Figure 4) indicate a nickel oxidation state of Ni^{+2}, which was also confirmed by iodometric titration. This indicates the total charge of the 8 anions is -13, indicating a stoichiometry of $La_3Ni_2O_5F_3$.

In addition, thermogravimetric data collected while heating $La_3Ni_2O_5F_3$ under a 10:90 H_2:N_2 gas mixture resulted in a mass loss of 4.95% (Figure S11). X-ray diffraction data collected from the products of the process indicate sample decomposed to a mixture of LaOF + Ni. Thus, a mass loss of 4.95% is consistent with an initial composition $La_3Ni_2O_{5.07(5)}F_{2.93(5)}$.

Bond valence sums calculated for the anion sites using parameters for O^{2-} and F^- (Table S5) indicate that fluoride ions occupy the apical and interstitial sites, and oxygen occupies the bridging and equatorial sites.

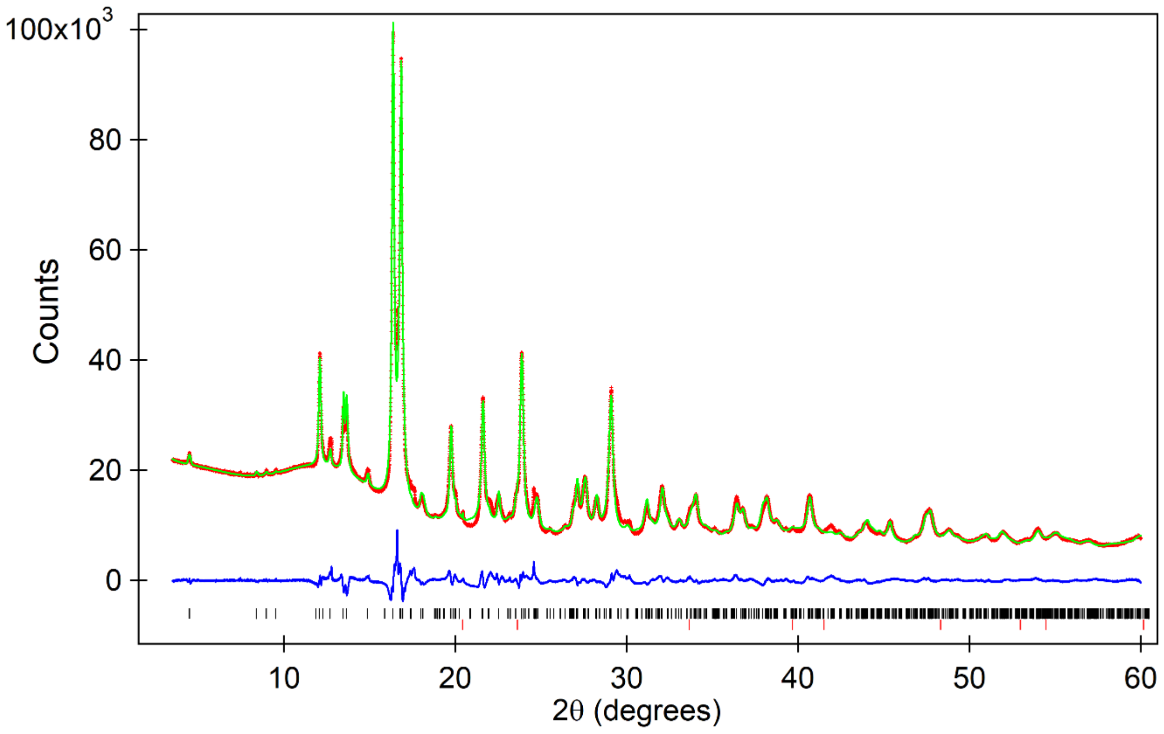

Figure S8. Observed, calculated and difference plots from the structural refinement of a *Pbcm* symmetry model against SXRD data collected from $La_3Ni_2O_5F_3$. Black ticks correspond to the majority phase and red ticks to LiF.

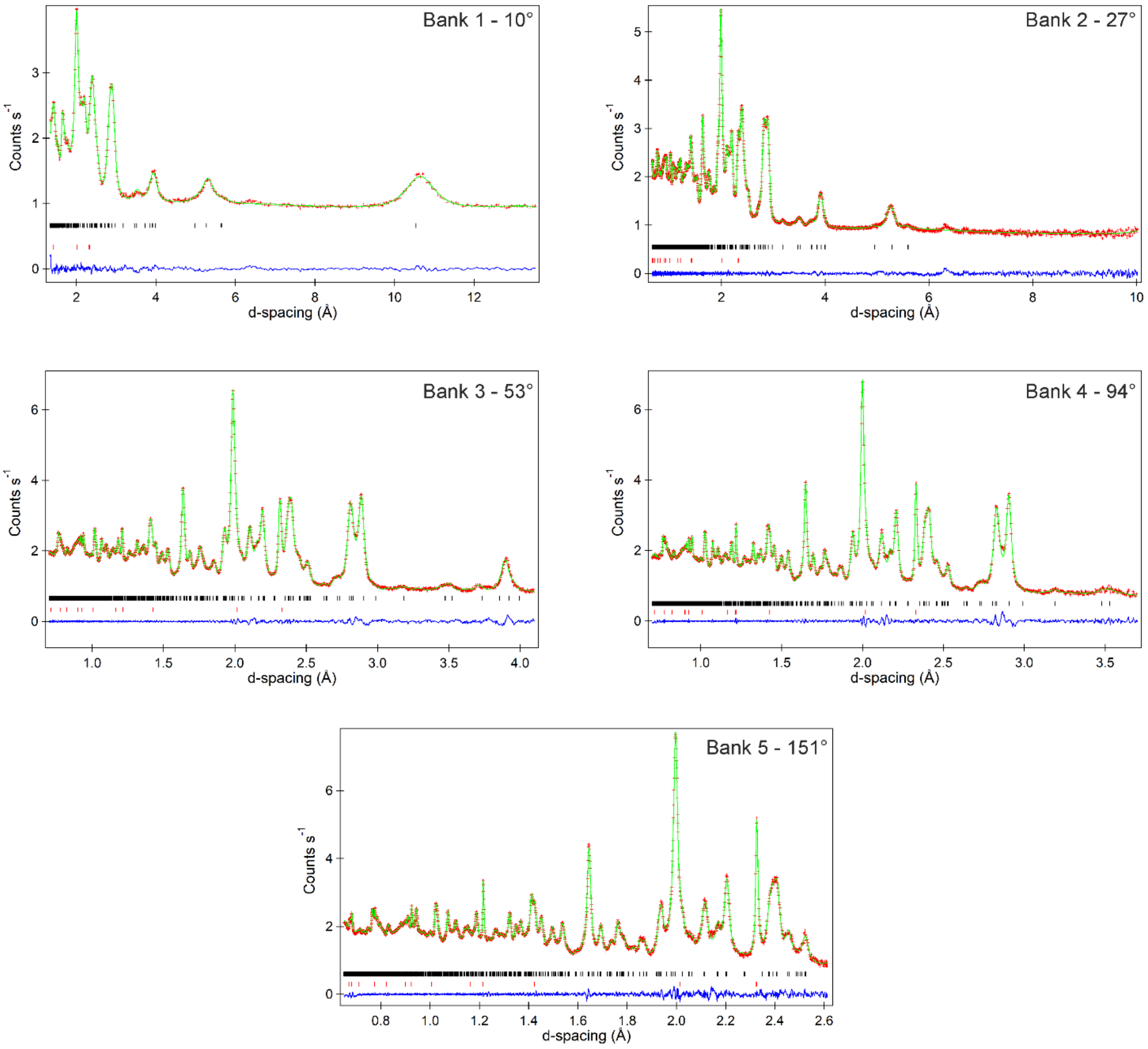

Figure S9. Observed, calculated and difference plots from the structural refinement of a *Pbcm* symmetry model against NPD data collected from $La_3Ni_2O_5F_3$ using the 5 detector banks of the POLARIS instrument at room temperature. Black ticks correspond to the majority phase, red ticks correspond to LiF.

Cation	Anion	Bond length (Å)
La(1)	O(1)	2.57(3)
	O(1)	2.809(19)
	O(1)	2.975(19)
	O(2)	2.89(2) × 2
	O(3)	2.507(19) × 2
	O(3)	2.78(2) × 2
La(2)	O(2)	2.22(3)
	O(2)	2.52(3)
	O(3)	2.54(2)
	F(1)	2.479(11)
	F(1)	2.59(4)
	F(1)	2.783(11)
	F(2)	2.399(19)
	F(2)	2.620(18)
Ni(1)	O(1)	1.99(3)
	O(2)	2.049(11)
	O(2)	2.085(11)
	O(3)	1.921(14)
	O(3)	2.163(14)
	F(1)	2.39(3)

Table S4. Selected bond lengths from the refined structure of $La_3Ni_2O_5F_3$.

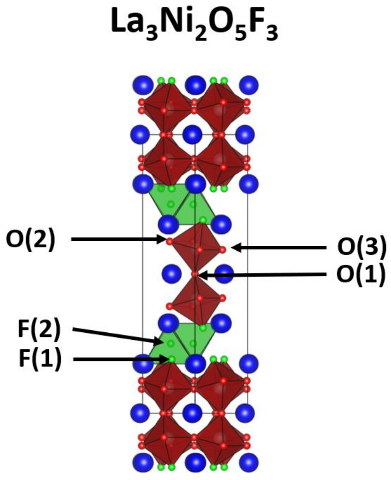

Figure S10. Crystal structure of $La_3Ni_2O_5F_3$ viewed down the [110] direction.

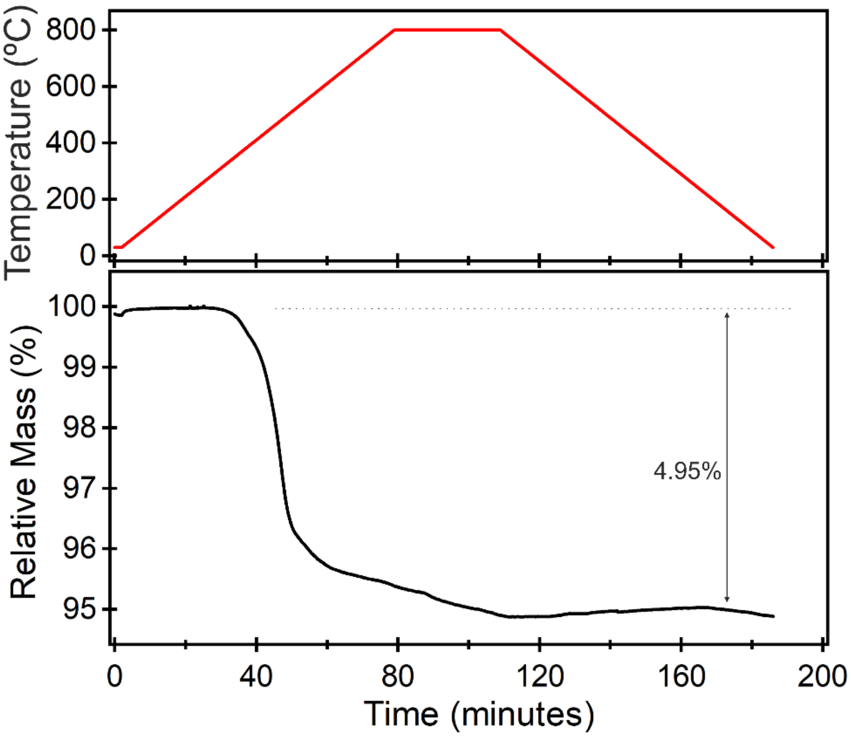

Figure S11. Thermogravimetric data collected while heating $La_3Ni_2O_5F_3$ under a 10:90 H_2:N_2 gas flow. X-ray diffraction data indicate the sample is converted to a mixture of LaOF + Ni, indicating an initial composition of $La_3Ni_2O_{5.07(5)}F_{2.93(5)}$.

$La_3Ni_2O_5F_3$	BVS F	BVS O	Assignment
Apical	**1.12**	1.35	Fluorine
Bridging	1.41	**1.76**	Oxygen
Equatorial O3	1.6	**1.94**	Oxygen
Equatorial O4	1.46	**1.77**	Oxygen
Tetrahedral	**1.27**	1.54	Fluorine

Table S5. Results of the bond valence sum calculations of the different anion sites in $La_3Ni_2O_5F_3$ assuming they are occupied by either oxygen or fluorine.

5. Characterisation of $La_3Ni_2O_5F$

SXRD and NPD data collected from the product of reaction between $La_3Ni_2O_5F_4$ and LiH at 250 °C could be indexed using a tetragonal cell (*a* = 3.983 Å, *c* =19.296 Å) with reflection conditions consistent with space group *I*4/*mmm* (#139) These parameters and space group are consistent with a T' type structure analogous to that of $La_3Ni_2O_6$, so a structural model based on this phase was refined simultaneously against both the NPD and SXRD data. Close inspection of the diffraction data sets revealed additional diffraction reflections attributable to LiF and LaOF, so these were added to the model as secondary phases. During the refinement, the SXRD data required a much higher concentration of these secondary phases compared to the NPD data, to achieve a good fit. We interpret this observation as indicating that these secondary phases exist as small particles on the surface of the sample, due to the slight surface sensitivity of SXRD data compared to NPD data. We therefore quote the phase fractions derived from the NPD data as these are more representative of the overall sample composition. The anion occupancies were refined to ensure that all sites were fully occupied.

In the final refinement cycle lattice parameters of all phases was allowed to refine, along with the atomic positional and displacement parameters of $La_3Ni_2O_5F$. The atomic positional parameters of LiF and LaOF were fixed at their literature values, and a single displacement parameter was refined for all the atoms in the secondary phases. The peak shape parameters of $La_3Ni_2O_5F$ were refined along with a separate combined set of peak shape parameters for LiF and LaOF (this was required due to the poor crystallinity of the secondary phases). In addition, a Stevens *hkl* dependent broadening term, which broadens reflections with a non-zero l component was also added to the model, for the majority phase. Instrumental zero-point and background parameters (Fourier cosine series) and the neutron absorption coefficients were also refined. As noted above separate phases fractions were refined for the NPD and SXRD data, with the values from the NPD data listed in Table 2. Full details for the refined structural parameters are described in Table 2 in the main text, with selected bond lengths given in Table S6 and plots of the data in Figure 5 in the main text and Figure S12 and S13.

The diffraction data indicate an overall composition of $La_3Ni_2(O/F)_6$. XANES data (Figure 4) indicate a nickel oxidation state of Ni^{1+}. This indicates the total charge of the 8 anions is -11, indicating a stoichiometry of $La_3Ni_2O_5F$.

In addition, thermogravimetric data collected while heating $La_3Ni_2O_5F_3$ under a 10:90 H_2:N_2 gas mixture resulted in a mass loss of 2.2% (Figure S15). X-ray diffraction data collected

from the products of the process indicate sample decomposed to a mixture of LaOF + La_2O_3 Ni. Thus, a mass loss of 2.2% is consistent with an initial composition $La_3Ni_2O_{5.04(5)}F_{0.96(5)}$ when the presence of LiF and LaOF secondary phases are taken into account.

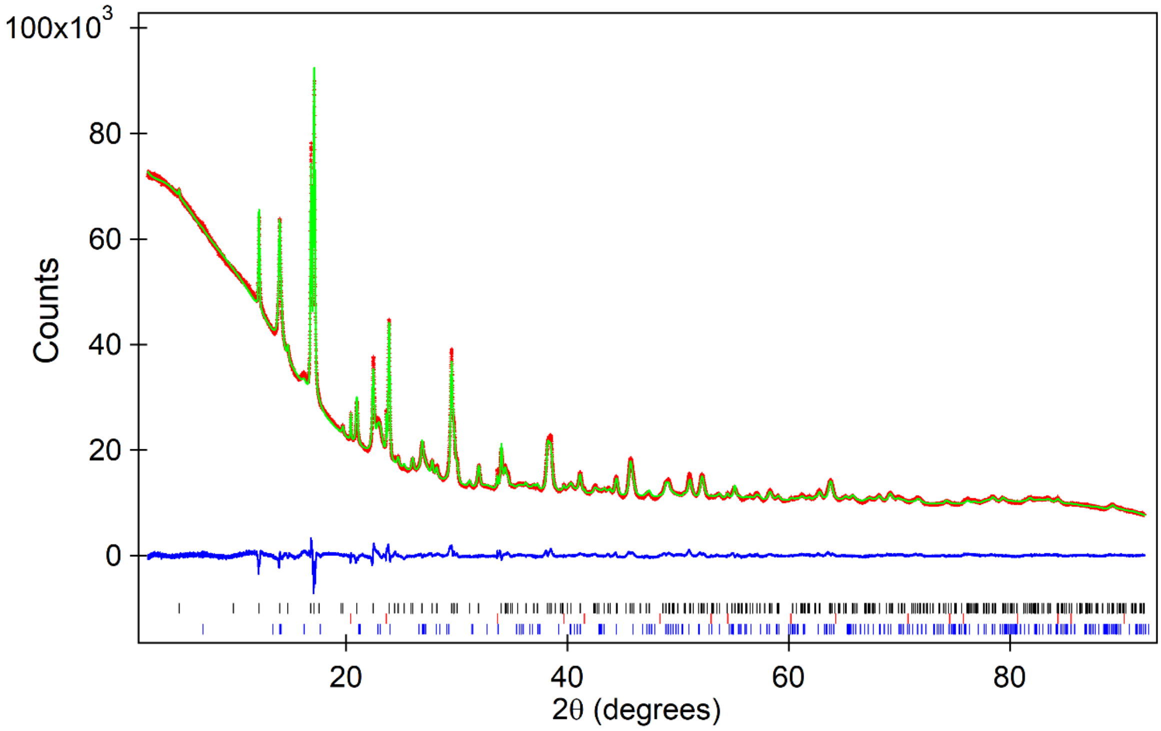

Figure S12. Observed, calculated and difference plots from the structural refinement of a *I*4/*mmm* symmetry model against SXRD data collected from $La_3Ni_2O_5F$. Black ticks correspond to the majority phase, red ticks to LiF and blue ticks LaOF.

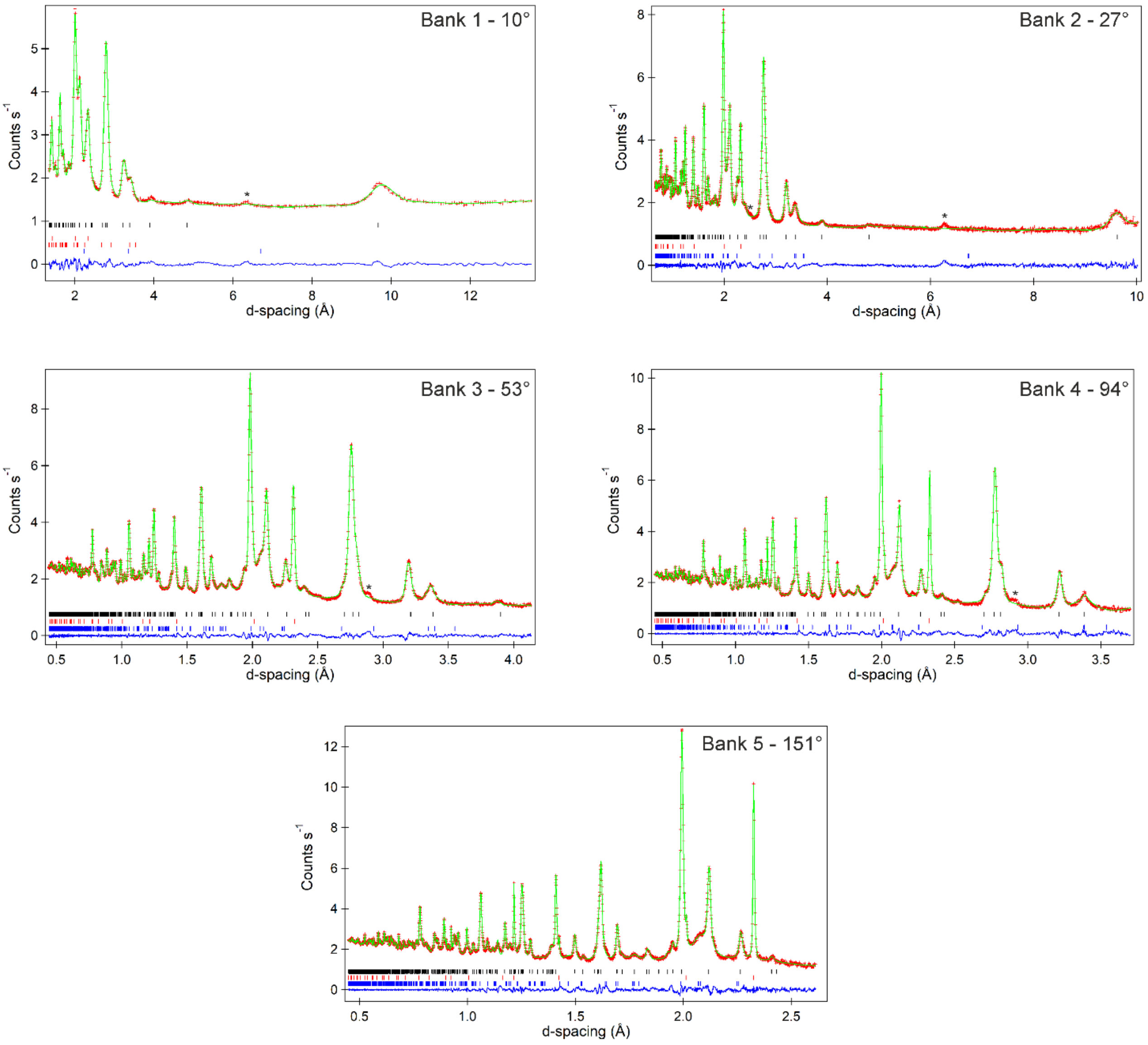

Figure S13. Observed, calculated and difference plots from the structural refinement of a *I*4/*mmm* symmetry model against NPD data collected from $La_3Ni_2O_5F$ using the 5 detector banks of the POLARIS instrument at room temperature. Black ticks correspond to the majority phase, red correspond to LiF and blue ticks correspond to LaOF. Unindexed peaks at d ~ 2.9 Å and ~6.2 Å, marked with * are an unidentified LaO_xF_y secondary phase.

Cation	Anion	Bond length (Å)
La(1)	O(1)	2.604(3) × 8
La(2)	O(1)	2.668(4) × 4
	O/F(2)	2.419(4) × 4
Ni(1)	O(1)	1.9923(9) × 4

Table S6. Selected bond lengths from the refined structure of $La_3Ni_2O_5F$.

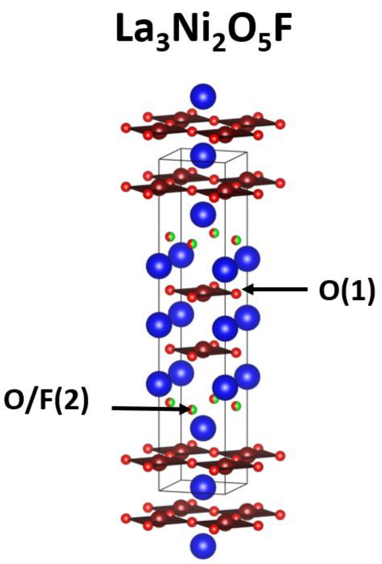

Figure S14. Crystal structure of $La_3Ni_2O_5F$.

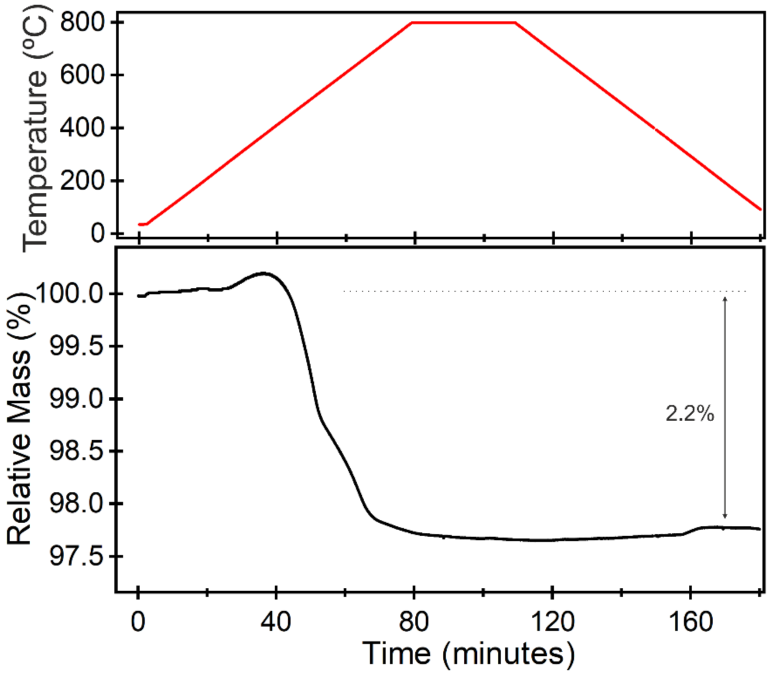

Figure S15. Thermogravimetric data collected while heating $La_3Ni_2O_5F$ under a 10:90 H_2:N_2 gas flow. X-ray diffraction data indicate the sample is converted to a mixture of LaOF + La_2O_3 + Ni, indicating an initial composition of $La_3Ni_2O_{5.04(5)}F_{0.96(5)}$ when the presence of LiF and LaOF secondary phases are taken into account.

6. Magnetic characterisation

Procedure used to measure the magnetization of samples containing elemental nickel:

The magnetization of elemental Ni is observed to saturate in applied magnetic fields of more than 2 T. Thus, the paramagnetic susceptibility of a bulk sample can be measured in the presence of elemental Ni impurities by measuring the gradient of magnetization-field isotherms in applied fields larger than 2.5 T, as shown in Figure S22. To this end the magnetization of samples was measured in a series of 5 fields between 3 T and 5 T. The magnetization vs. field data were fitted to a linear function, the gradient of which is the paramagnetic susceptibility of the bulk sample and the intercept is the saturated ferromagnetic moment of the sample. Data points with large errors were excluded from fits. All fits had at least 4 data points. This procedure was repeated at 5 K intervals between 5 K and 300 K to measure the temperature dependent susceptibility of samples.

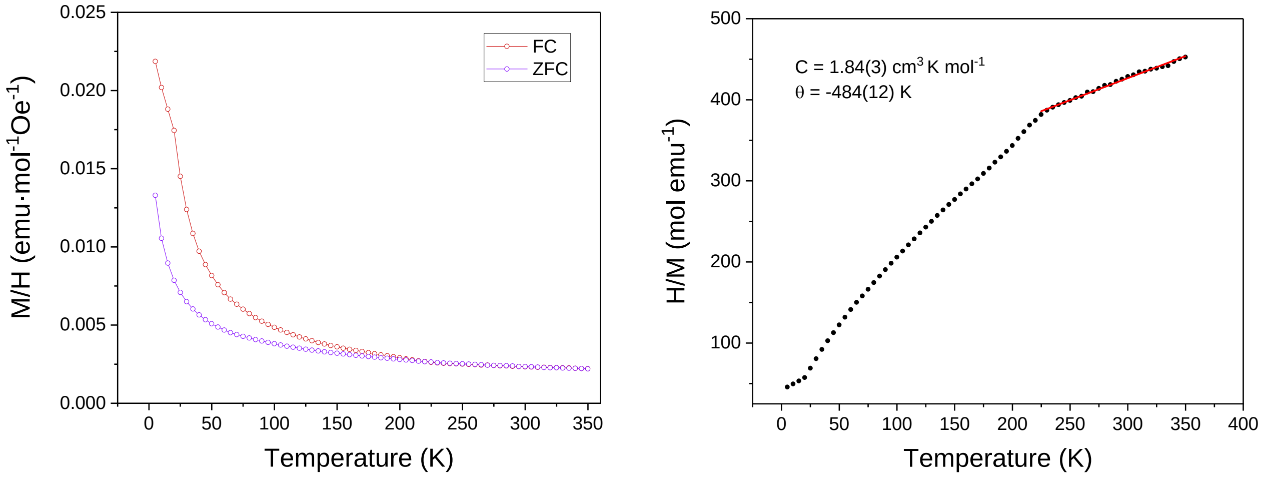

Figure S16. Field cooled and zero field cooled magnetisation data collected from $La_3Ni_2O_5F_4$ as a function of temperature in an applied field of 100 Oe (left) and corresponding reciprocal curve fitted to the Curie-Weiss law (right).

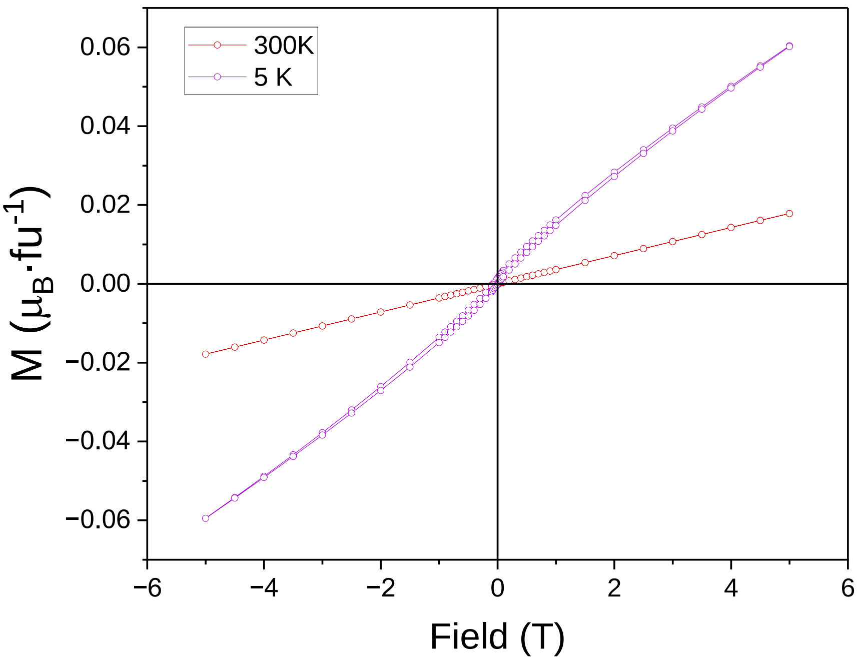

Figure S17. Magnetization data collected from $La_3Ni_2O_5F_4$ at 300 K and 5 K, as a function of applied field. The data at 5 K were collected after field cooling from 300 K in an applied field of 50 000 Oe.

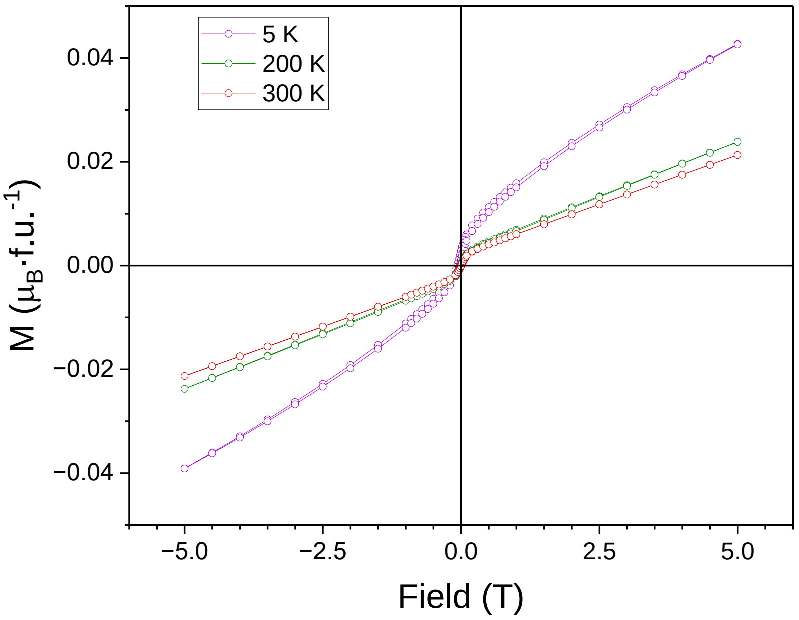

Figure S18. Magnetization data collected from $La_3Ni_2O_5F_3$ at 300 K, 200 K and 5 K, as a function of applied field. The data at 5 K and 200 K were collected after field cooling from 300 K in an applied field of 50 000 Oe.

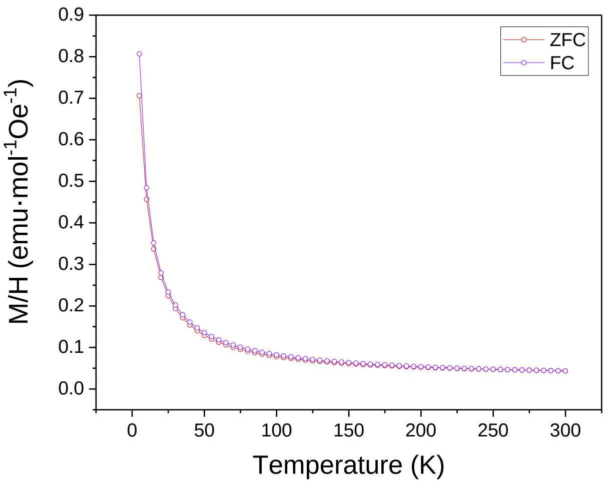

Figure S19. Field cooled and zero field cooled magnetisation data collected from $La_3Ni_2O_5F$ as a function of temperature in an applied field of 100 Oe.

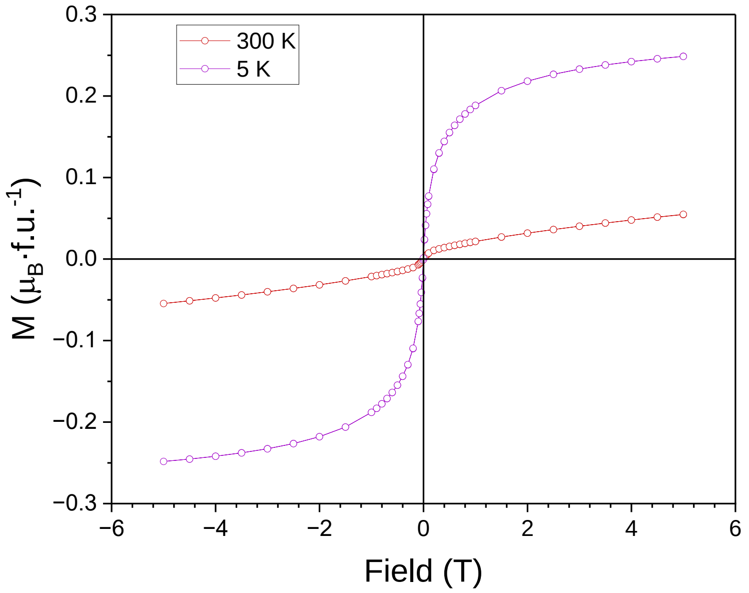

Figure S20. Magnetization data collected from $La_3Ni_2O_5F$ at 300 K and 5 K, as a function of applied field. The data were collected at 5 K after field cooling from 300 K in an applied field of 50 000 Oe.

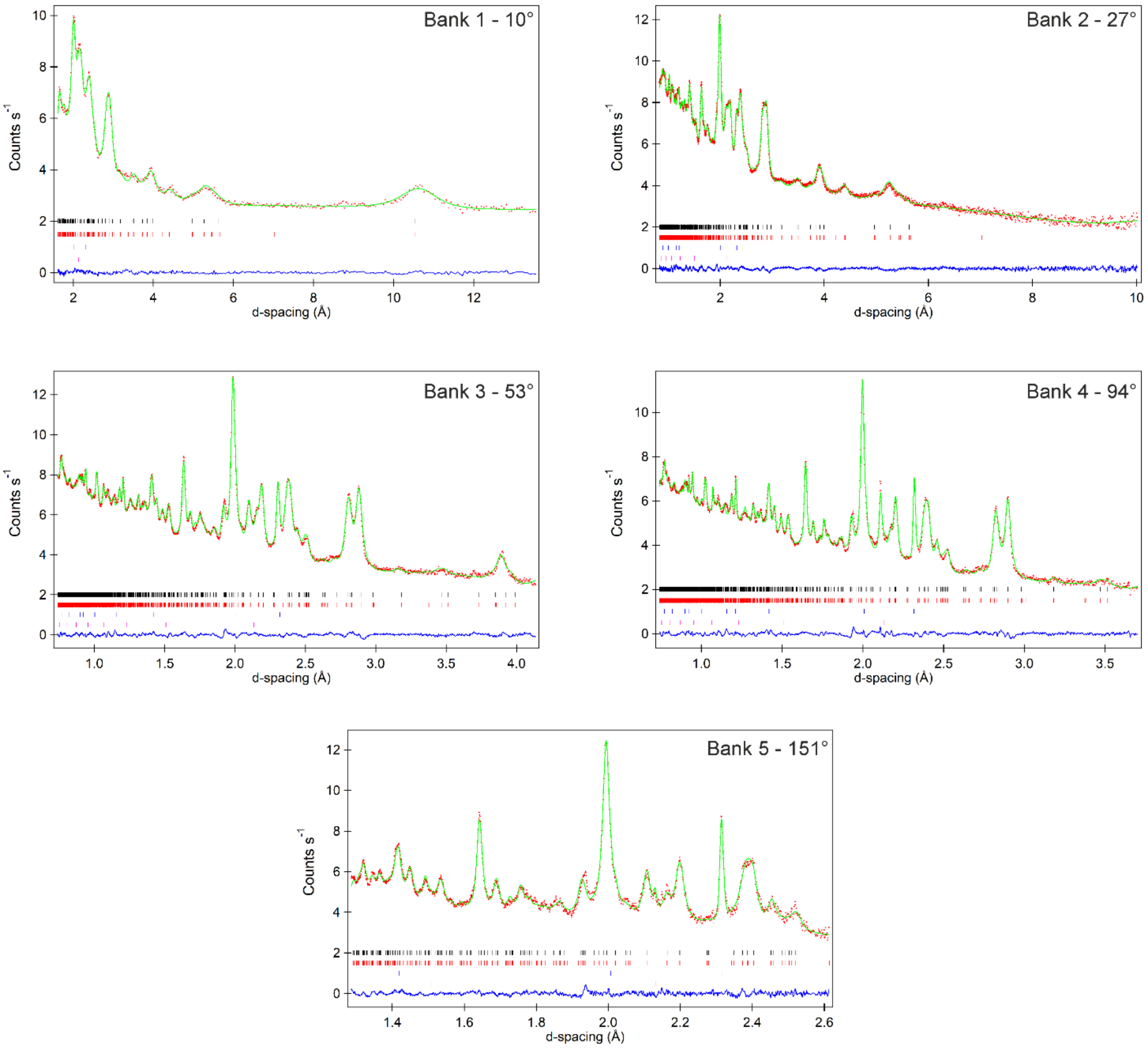

Figure S21. Observed, calculated and difference plots from the structural refinement of a *Pb'c'm* symmetry model against NPD data collected from $La_3Ni_2O_5F_3$ using the 5 detector banks of the POLARIS instrument at 10 K. Green ticks correspond to the nuclear scattering of the majority phase, purple ticks correspond to its magnetic scattering peaks, black ticks correspond to LiF and orange ticks correspond to the vanadium can.

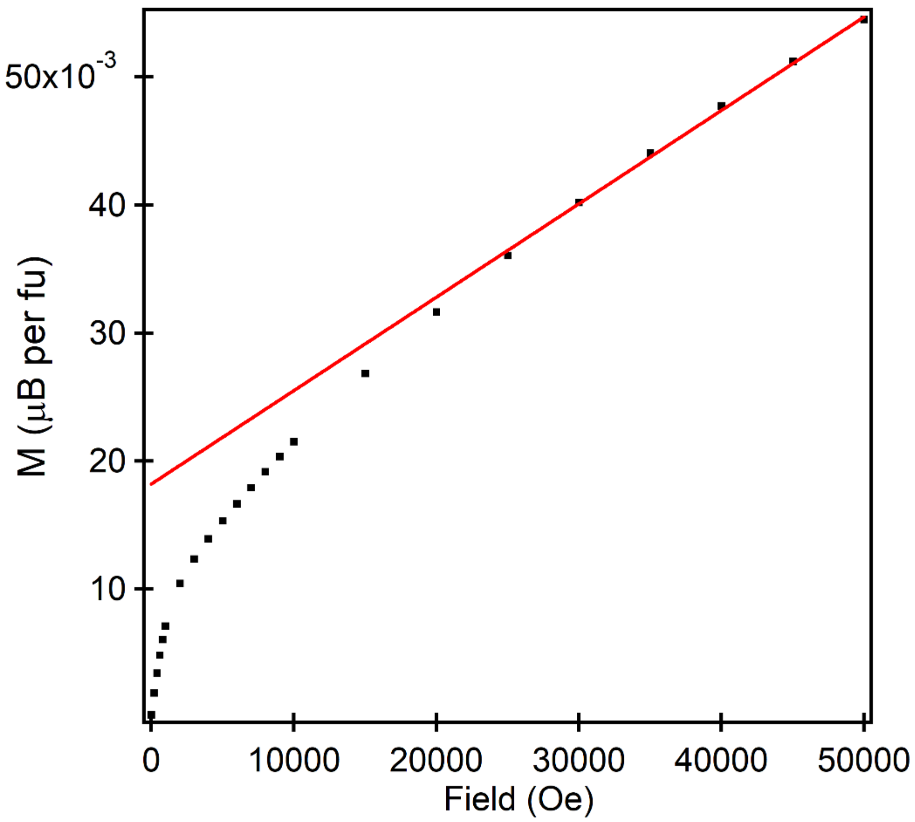

Figure S22. Magnetization of $La_3Ni_2O_5F$ measured as a function of applied field at 300 K. A linear fit to high-field region (H > 30000 Oe) yields a gradient which is the paramagnetic susceptibility of the sample, and an intercept which is the saturated ferromagnetic moment of the sample.

References

1) Jorgensen, J. D.; Dabrowski, B.; Pei, S.; Richards, D. R.; Hinks, D. G. Structure of the Interstitial Oxygen Defect in $La_2NiO_{4+\delta}$. *Phys. Rev. B* **1989**, *40* (4), 2187–2199. https://doi.org/10.1103/PhysRevB.40.2187.

23) Zhu, T.; Khalsa, G.; Havas, D. M.; Gibbs, A. S.; Zhang, W.; Halasyamani, P.; Benedek, N. A.; Hayward, M. A. Cation Exchange as a Mechanism to Engineer Polarity in Layered Perovskites. *Chem. Mater.* **2018**, *30*, 8915–8924.
